# Molecular Engineering of the Kinetic Barrier in Seeded Supramolecular Polymerization

**DOI:** 10.1021/jacs.2c10482

**Published:** 2023-02-24

**Authors:** Qin Huang, Nicolas Cissé, Marc C. A. Stuart, Yaroslava Lopatina, Tibor Kudernac

**Affiliations:** † Stratingh Institute for Chemistry, University of Groningen, Nijenborgh 4, 9747 AG Groningen, The Netherlands; ‡ Groningen Biomolecular Sciences and Biotechnology Institute, University of Groningen, Nijenborgh 7, 9747 AG Groningen, The Netherlands

## Abstract

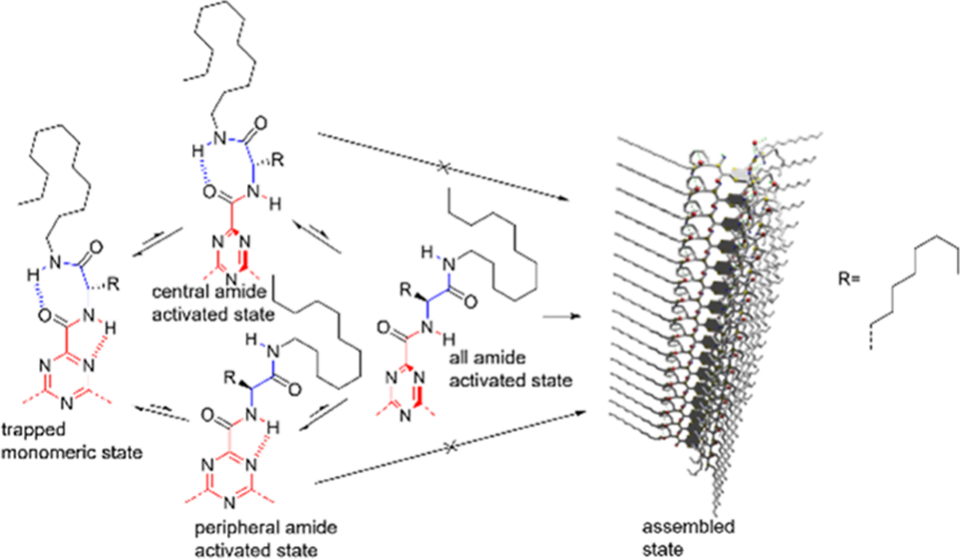

Seeded supramolecular polymerization (SSP) is a method that enables the controlled synthesis of supramolecular structures. SSP often relies on structures that are capable of self-assembly by interconverting between intramolecular and intermolecular modes of hydrogen bonding, characterized by a given kinetic barrier that is typically low. The control of the polymerization process is thus limited by the propensity of the hydrogen bonds to interconvert between the intramolecular and intermolecular modes of binding. Here, we report on an engineering of the polymerization kinetic barriers by sophisticated molecular design of the building blocks involved in such SSP processes. Our designs include two types of intramolecular hydrogen-bonded rings: on one hand, a central triazine tricarboxamide moiety that prevents self-assembly due to its stable intramolecular hydrogen bonds and on the other hand, three peripheral amide groups that promote self-assembly due to their stable intermolecular hydrogen bonds. We report a series of molecules with increasing bulkiness of the peripheral side chains exhibiting increasing kinetic stability in the monomeric form. Owing to the relative height of the barrier, we were able to observe that the rate constant of seeding is not proportional to the concentration of the seeds used. Based on that, we proposed a new kinetic model in which the rate-determining step is the activation of the monomer, and we provide the detailed energy landscape of the supramolecular polymerization process. Finally, we investigated the hetero-seeding of the building blocks that shows either inhibition or triggering of the polymerization.

## Introduction

Supramolecular polymerization is a spontaneous process that relies on strong noncovalent interactions between monomers.^1,2^ Such spontaneity hampers the control over the polymerization process. In the case of seeded supramolecular polymerization (SSP),^3,4^ the role of seeds is to skip the nucleation step in the course of the process so that the supramolecular polymers’ average length, size distribution, and composition sequence can be controlled.^3,5−8^

Various design characteristics must be met to reach a system exhibiting effective seeded supramolecular polymerization.^4^ From the thermodynamic point of view, the polymerization process should be cooperative,^1^ i.e., during the formation of a nucleus, the addition of an individual monomer should be an unfavorable process in comparison to the elongation of an already existing polymer strand. From the kinetic point of view, the energy barrier of the transition from the monomeric state to the self-assembled state should be high enough to produce a kinetically trapped monomeric state with a controllable lifetime. It is reported in the literature that kinetic control over the supramolecular polymerization can be achieved through pathway complexity. In such an approach, the monomers are trapped within off-pathway aggregates that must disassemble prior any polymerization of the on-pathway aggregates, which happens only when the on-pathway nuclei are presented.^3,7,9^ An alternative way to achieve kinetic control over the polymerization process is to design monomers with kinetically trapped inactive conformations. Würthner and co-workers reported a perylene bisimide (PBI) derivative in which the monomeric state is trapped by the intramolecular hydrogen bonding of the amide groups.^10^ Aida and co-workers reported a corannulene derivative containing five amide groups. In MCH, the monomeric state is effectively trapped by a fivefold intramolecular hydrogen bond. The addition of a methyl-substituted corannulene derivative, whose carbonyl groups can interact with the trapped monomers as hydrogen bond acceptors, triggers the opening of the intramolecular hydrogen bonds progressively, thus enabling polymerization.^6^ There are a few more examples of seeded supramolecular polymerization controlled by the conversion from intra- to intermolecular hydrogen bonding of amide groups^11−13^ and even an example where this strategy is combined with ligand binding to an adjacent group.^14^ Indeed in some cases, monomers can form off-pathway aggregates while maintaining their intermolecular hydrogen-bonded trapped conformation.^15−18^ Instead of carbonyl groups, a fluorinated hexane was recently reported as a new type of intramolecular hydrogen bond acceptor to create a trapped monomeric state for SSP.^8^

**Figure 1:**
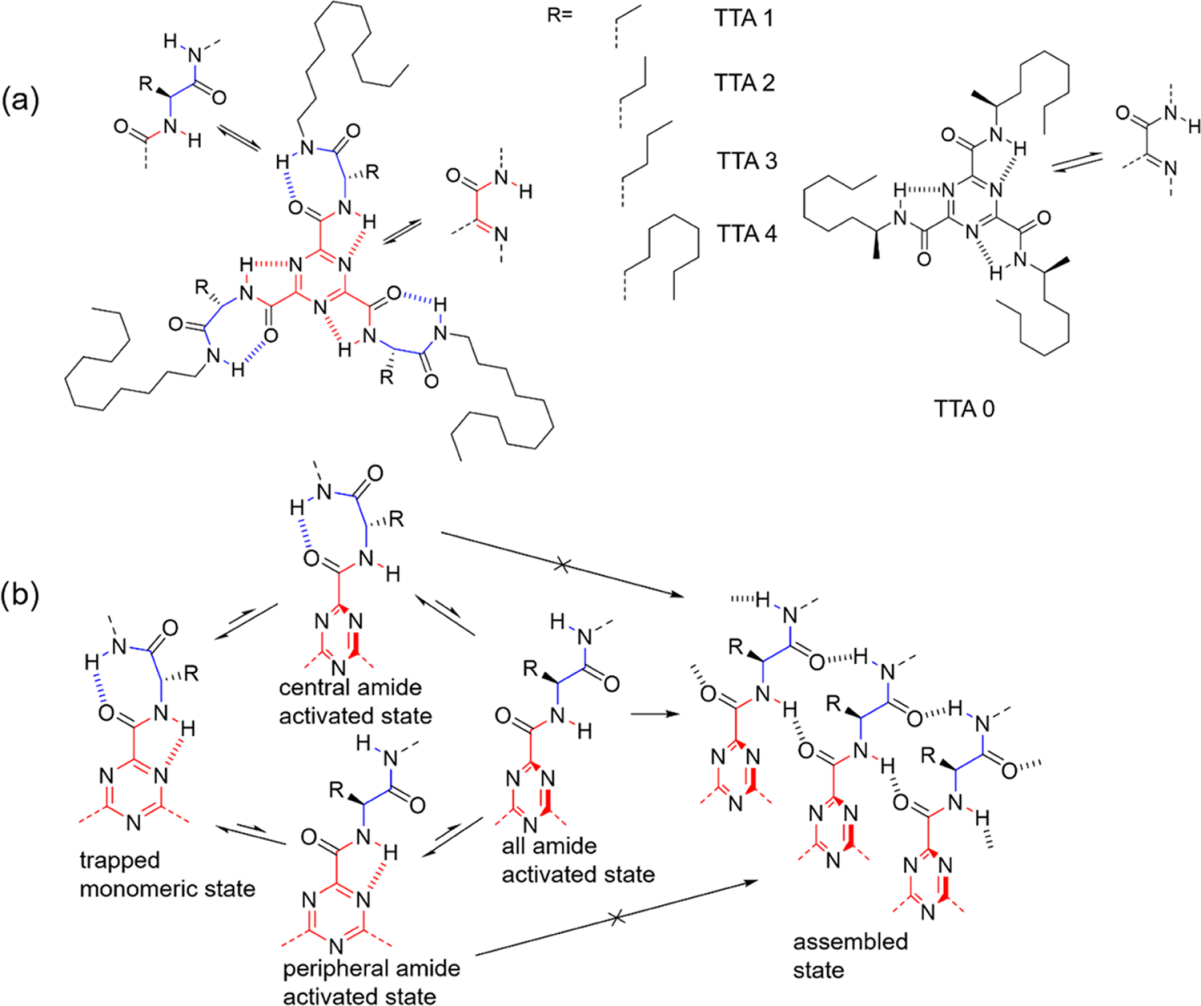
(a) Molecular structures of TTA 1–4 (left) and TTA 0 (right). (b) Proposed hydrogen bond changes leading to self-assembly, both the central and the peripheral amide groups must be activated to allow supramolecular polymerization.

To achieve SSP, our molecular design strategy relies on the interconversion of hydrogen bonds between intra- and intermolecular bonded states. Hence, hydrogen bonds are both responsible for the high kinetic barrier and for the intermolecular binding. In such an approach, the intramolecular hydrogen bonding is necessarily weaker than the intermolecular hydrogen bonding, which often limits the height of the kinetic barrier and thus affects the SSP process. Here, we demonstrate that the key strategy to overcome this limitation consists in introducing multiple hydrogen-bonded trapped states within the same molecule. Inspired by the cooperative mechanism of benzene-1,3,5-tricarboxamide (BTA) supramolecular polymerization,^19^ we designed a series of triazine-1,3,5-tricarboxamide (TTA) derivatives devised to facilitate the intramolecular hydrogen bonding of the central amide groups with the nitrogen atoms of the triazine ring as the hydrogen bond acceptors (Figure 1). The other three peripheral amide groups can form seven-membered rings through intramolecular hydrogen bonding; the rings must first open to allow the polymerization to take place. Our strategy to achieve a high kinetic barrier is to synchronize the transition from intra- to intermolecular hydrogen bonds of both types of hydrogen-bonding motives. For each arm of the TTA 1–4 series, we expect that neither the central amide groups nor the peripheral amide groups can independently switch to their intermolecular hydrogen-bonded states (Figure 1b). This means that the intermolecular hydrogen bonding can only be realized after the opening of both type of intramolecular hydrogen-bonded rings at the same time.

## Results and Discussion

### Molecular Design and Mechanism

To get an insight into the self-assembly mechanism of TTA 1–4, the energy landscape of TTA 0 was investigated first. The design of TTA 0 includes the same central amide groups as TTA 1–4 but does not include any peripheral amide groups (Figure 1a). The ^1^H NMR spectra of a 4.78 mM solution of TTA 0 at 298 K in MCH-d_14_ reveal an amide peak with a shape that is similar to the one in CDCl_3_, in which TTA 0 is molecularly dissolved (Figure S1a). This suggests that TTA 0 remains in the monomeric state after fast cooling to room temperature from the MCH boiling point. Further on, a 200 μM solution of TTA 0 in MCH was cooled at a rate of 10 K min^–1^ from 353 to 298 K, and the resulting CD spectrum confirms that TTA 0 still remains in its monomeric state (Figure S1b). The CD signal corresponding to the monomeric state is stable during 5 days at 298 K. Together with the NMR experiments, these results suggest that TTA 0 at 200 μM is more stable in its monomeric state. Then, the IR spectra of TTA 0 and BTA, a control building block, which is unable to form any intramolecular hydrogen bonds, were investigated in both CHCl_3_ (Figure S2a) and MCH (Figure S2b). The N–H stretch of TTA 0 is similar in these two solutions, while the N–H stretch of BTA shifts toward lower wavenumbers from CHCl_3_ to MCH, suggesting that TTA 0 is intramolecularly hydrogen-bonded in CHCl_3_ and MCH.

For TTA 1–4, the oxygen atoms of the central amide groups can form hydrogen bonds with the hydrogen atoms of the peripheral amide groups, thus forming the peripheral seven-membered rings (Figure 1a, left).^13,14^ The R group positioned in between the two amides directly influences the propensity of the monomer to polymerize because the molecule reaches its activated state through the opening of the peripheral seven-membered rings bearing R (Figure 1b). The larger the R, the narrower, the range of the R–C–N–C dihedral angle possibilities. This is due to the collision between the R group and the amide groups on its two sides.^20^ Let us consider the case of the peripheral seven-membered ring opening, while the central amide groups remain in their intramolecularly hydrogen-bonded state. The R group is directed out of the plane of the triazine and poses a steric hindrance to the intermolecular face-to-face interactions between two monomers. For larger R groups, the distance between the interacting monomers exceeds the effective range of attractive interactions. Therefore, polymerization cannot take place when only the peripheral amide groups are activated. However, if the size of the R group is small enough, the activation of the peripheral amide only is sufficient to trigger supramolecular polymerization (Figure S3). Let us now consider the case when only the central amide groups are activated (Figure 1b). In this state, the binding energy is too weak to result in any thermodynamically stable supramolecular polymers, like for TTA 0. These two design principles ensure that the self-assembly can only take place after both the central and the peripheral amides groups are activated. Indeed, in the fully activated state, the R groups are reoriented almost parallel to the plane of the triazine; thus, two monomers can get close together, allowing the formation of intermolecular hydrogen bonds. Such double activation increases the energy of the activated intermediate, resulting in the trapping of the monomeric state and yielding a self-assembly system with a high kinetic barrier (Figure 1b).

### Temperature-Dependent Measurements

**Figure 2:**
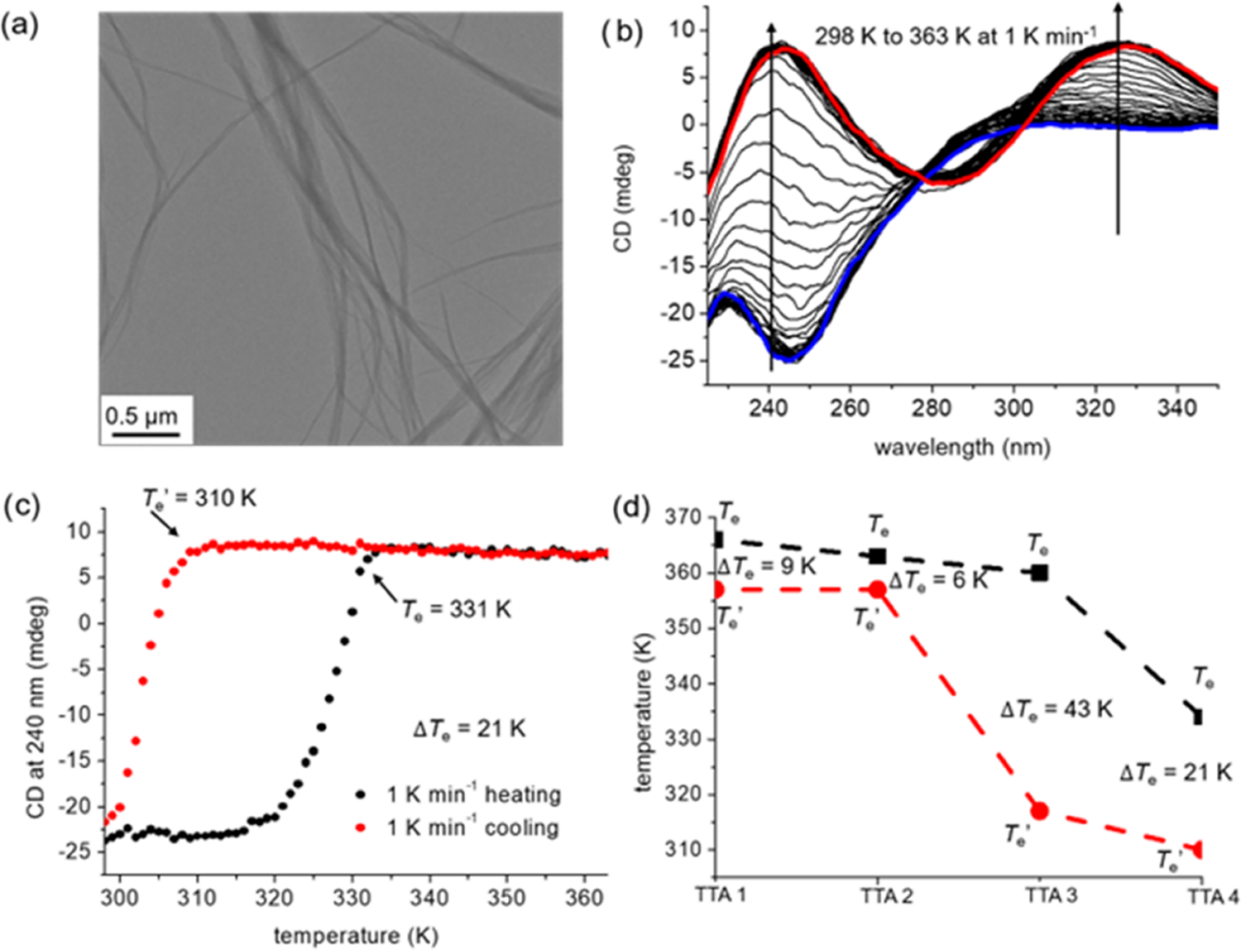
(a) TEM of TTA 4 assembled in MCH at 100 μM at 298 K and dried. (b) CD spectrum of TTA 4 at 100 μM in MCH upon heating at 1 K min^–1^. (c) CD at 240 nm of TTA 4 at 100 μM in MCH upon heating and cooling at 1 K min^–1^. (d) Critical elongation temperature upon heating (*T*_e_) and cooling (*T*_e_′) process, and Δ*T*_e_ = *T*_e_ – *T*_e_′.

TEM microscopy shows the formation of supramolecular fibers at 298 K with similar morphologies for all four compounds in the series (Figures 2a and S4g–i). AFM microscopy also shows a similar fiber morphology (Figure S5). The self-assembly of TTA 1–4 in methylcyclohexane (MCH) was investigated by temperature-dependent CD spectroscopy (Figures 2b,c and S4) and UV–vis spectroscopy (Figure S6). All of the compounds have a similar CD spectrum in both the assembled state below 300 K and the monomeric state obtained by heating to 363 K for TTA 4 and 368 K for TTA 1–3 (Figures 2b and S4a–c). To estimate the height of their polymerization kinetic barrier, the thermal hysteresis of their polymerization was investigated by heating and cooling cycles at 1 K min^–1^ (Figures 2c and S4d–f). The critical elongation temperatures are obtained by fitting the heating and cooling curves to the thermodynamic model proposed by Meijer et al.^22^ The critical elongation temperature *T*_e_ upon heating is higher than the critical elongation temperature *T*_e_′ upon cooling. The difference Δ*T*_e_ = *T*_e_ – *T*_e_′ contains the contribution from the polymerization kinetic barrier and other influences such as fragmentation propensity. Normally, the kinetic barrier dominates the Δ*T*_e_ and thus can be roughly estimated by measuring Δ*T*_e_ (Figure 2d). The larger Δ*T*_e_ means a higher kinetic barrier.^10^ The slightly higher Δ*T*_e_ value of TTA 1 with respect to TTA 2 (Figure 2d) is probably due to the decreased stability of the trapped state with the increasing size of R by reducing the flexibility of the seven-membered ring formed by intramolecular hydrogen bonding, while the transition states of TTA 1 and TTA 2 have a similar free energy (Figure S3b). The Δ*T*_e_ of TTA 3 is 43 K, which is significantly larger than the Δ*T*_e_ of TTA 1 (9 K) and TTA 2 (6 K). These large hysteresis differences confirm that the polymerization kinetic barrier of TTA 3 is higher than the kinetic barriers of TTA 1 and TTA 2 due to the increasing bulkiness of the R group. However, increasing the R group further results in a decrease of Δ*T*_e_ (TTA 4 has a Δ*T*_e_ of 21 K). On one hand, the longest R group in TTA 4 still plays its role in forming a system with a high kinetic barrier, but on the other hand, the longest R group destabilizes the fibers that are consequently easier to break into shorter segments, which in turn accelerates further growth and thus lower the Δ*T*_e_. Due to the strong influence of fiber fragmentation, the Δ*T*_e_ of TTA 4 measured in these conditions does not reflect on the real height of the kinetic barrier.

To further compare the real kinetic barriers of TTA 3 and TTA 4, hysteresis at a lower concentration (30 μM) was investigated (Figure S7) because at lower concentrations, the primary nucleation can occupy a larger ratio of the whole nucleation time and the influence of fragmentation can be reduced (discussion in Figure S7). After optimization of the heating and cooling rates to 0.2 K min^–1^ at this concentration(Figure S7a), the Δ*T*_e_ of TTA 4 (Figure S7b) and TTA 3 (Figure S7c) was 21 and 9 K, respectively. This result was obtained by reducing the influence of fiber fragmentation and is consistent with the assumption that TTA 4 has a higher kinetic barrier than TTA 3.

### Time-Dependent Measurements

**Figure 3:**
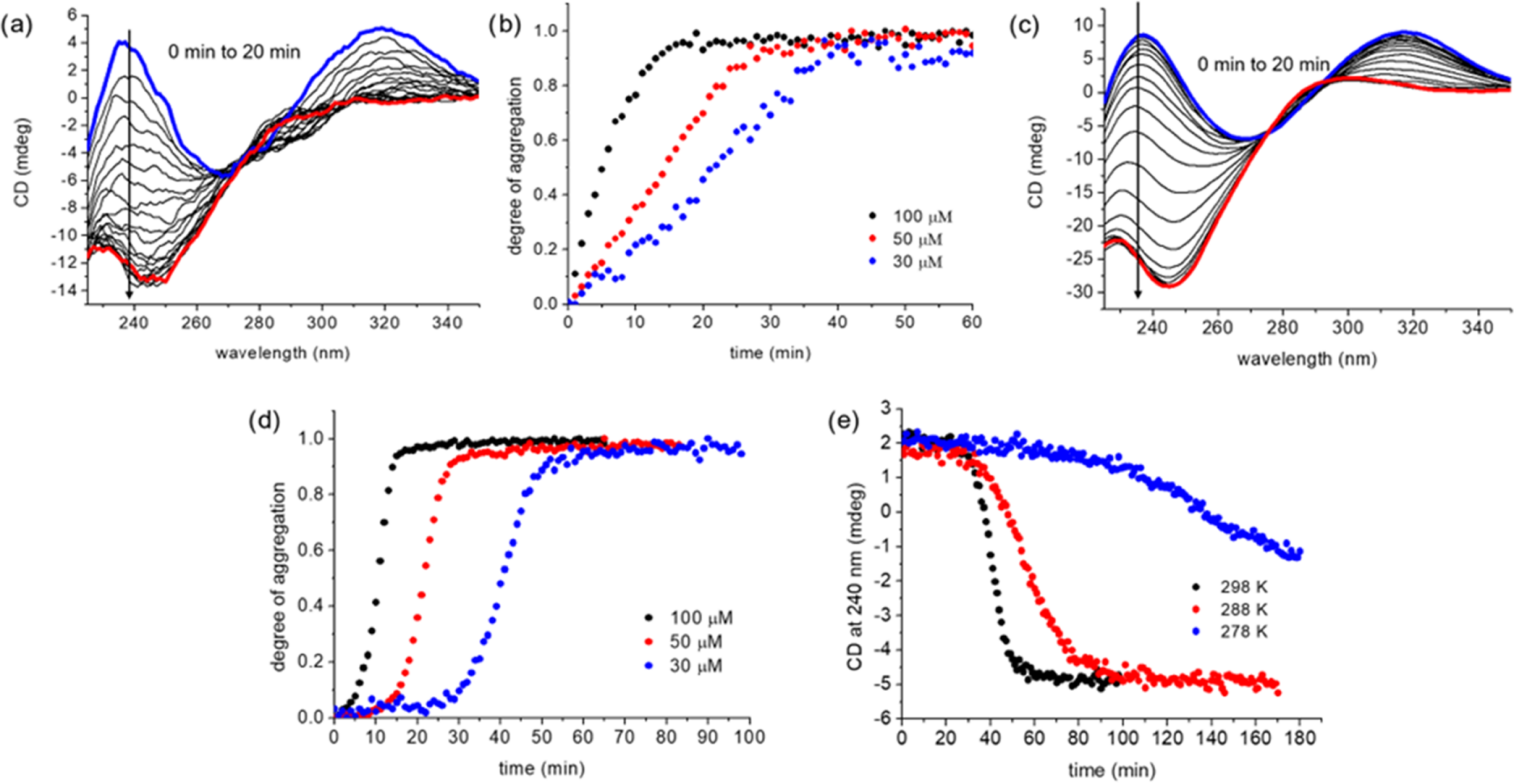
(a) CD of 100 μM solution of TTA 3 in MCH at 298 K after cooling from 368 K at the rate of 10 K min^–1^. (b) Degree of aggregation (normalized CD signal at 240 nm) for TTA 3 at 100 μM, 50 μM, and 30 μM in MCH measured at 298 K after fast cooling from 368 K at the rate of 10 K min^–1^. (c) CD of 100 μM solution of TTA 4 in MCH at 298 K after cooling from 358 K at the rate of 10 K min^–1^. (d) Degree of aggregation (normalized CD signal at 240 nm) for TTA 4 at 100 μM, 50 μM, and 30 μM in MCH measured at 298 K after fast cooling from 358 K at the rate of 10 K min^–1^. (e) Evolution of the CD signal at 240 nm of 30 μM TTA 4 in MCH at 298 K, 288 K, and 278 K after cooling from 358 K at the rate of 10 K min^–1^.

The kinetic stability of the monomeric state of TTA 3 and TTA 4 and their subsequent polymerization kinetics were investigated by CD spectroscopy. Typically, solutions of TTA 3 and TTA 4 in MCH were heated up to 368 and 358 K, respectively, for 5 min to reach their monomeric states. Then, these solutions were cooled down to 298 K at a rate of 10 K min^–1^. The CD spectrum immediately obtained after cooling TTA 3 (Figure 3a) is similar to the CD spectrum of TTA 3 in its monomeric state at 368 K (Figure S4c). This suggests that most of TTA 3 molecules are in their monomeric state immediately after cooling. To verify if this state is effectively a monomeric state and not an occasional off-pathway aggregated state with a similar CD spectrum, the degree of aggregation was monitored over time at different building block concentrations (Figure 3b). Typically, off-pathway aggregation is indicated by a slower rate of polymerization at higher building block concentration.^3,10,11,16,18^ Here, the rate of polymerization is slower at lower building block concentrations, which confirms that TTA 3 is effectively trapped in its monomeric state after cooling. The same goes for TTA 4 (Figure 3c,d).^10^ TTA 3 did not show any significant nucleation period even at a low concentration (30 μM), while TTA 4 showed a significant nucleation period with a longer nucleation time at a lower concentration. This also suggests that TTA 4 has a higher kinetic barrier than TTA 3. Further, the 30 μM solution of TTA 4 was cooled to various temperatures (278, 288, and 298 K, Figure 3e). At lower cooling temperatures, we observed longer nucleation times and slower polymerization rates. This differs from previous report, suggesting that the trapped monomeric state is more prone to nucleation at lower temperatures.^10^ TThis difference is attributed to the high kinetic barrier of TTA 4, which probably makes the rate determining step become the thermal activation of the trapped monomer, rather than the nucleation of the activated monomer. The self-assembly behavior after cooling was also investigated by UV–vis spectroscopy and confirmed these findings (Figure S8).

### Seeding Experiment and Modeling

**Figure 4:**
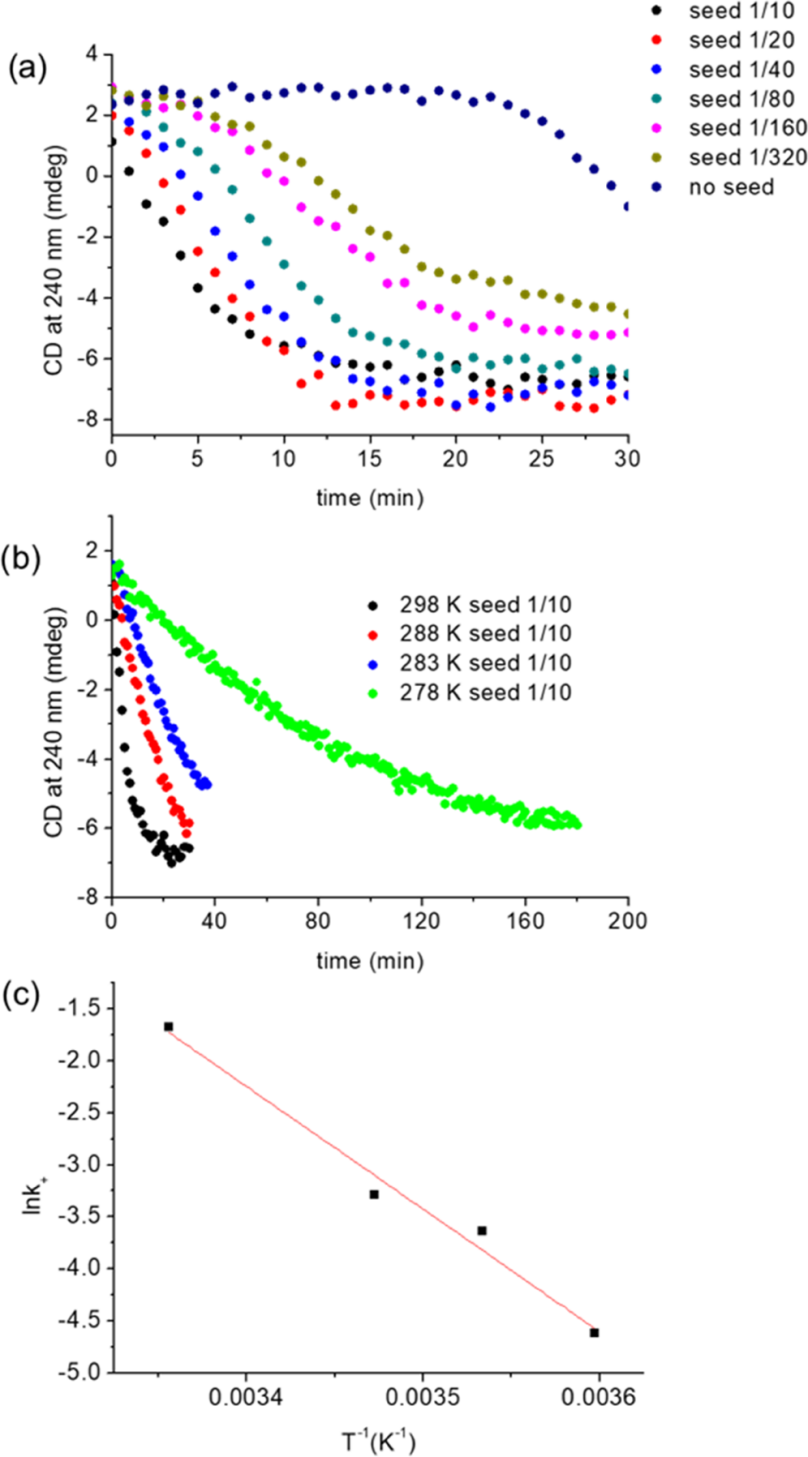
(a) Solution of TTA 4 in MCH at 298 K after cooling from 358 K at the rate of 10 K min^–1^ and with added seeds at various concentrations. The total concentration is 30 μM. (b) TTA 4 in MCH at 298 K, 288 K, 283 K, and 278 K after cooling at the rate of 10 K min^–1^ from 358 K with the seed ratio 1/10. The total concentration is 30 μM. (c) Arrhenius plot with *R*^2^ = 0.975. The kinetic barrier of TTA 4 is estimated as Δ*E*^#^ = 98 kJ mol^–1^.

TTA 4 was used at a concentration of 30 μM for the seeding experiments due to its convenient long nucleation time of more than 20 min at this concentration. TTA 4 seeds were prepared by sonication for 20 s, and the influence of sonication was investigated by UV–vis (Figure S9d), CD (Figure S9h), and TEM (Figure S10d). Various concentrations of seeds were added to the solutions of kinetically trapped monomers, while the overall concentration of 30 μM was kept constant. The ratio between the molecules present in the added seeds and in the monomeric state ranged from 1/10 to 1/320. We found that a higher proportion of seeds yields a higher rate of polymerization at a constant temperature of 298 K (Figure 4a). At lower ratios (1/320 and 1/160), the initial rates of polymerization are slower and gradually accelerating. The same acceleration occurs for the seeding at 288 K (Figure S11a) for a ratio of 1/80. This suggests that the concentration of seeds increases during the polymerization process, probably as a result of the breaking of the growing fibers due to the sterically destabilizing large R group of TTA 4. The seeding of TTA 4 was also investigated by DLS, which also supports the triggering of polymerization by TTA 4 seeds (Figure S12).

**Figure 5:**
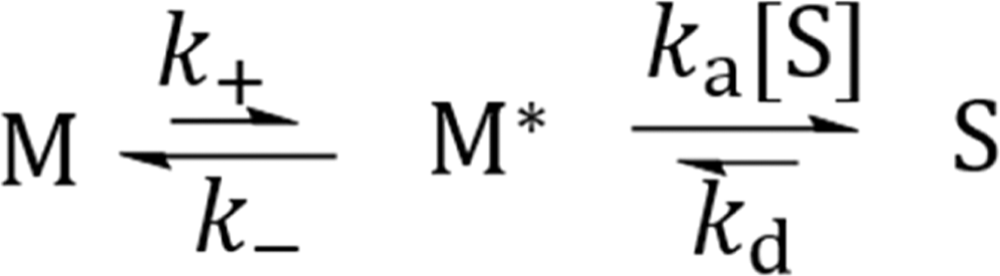
Proposed kinetic model for the seeded polymerization of TTA 4.

The rate of polymerization in the seeding experiments is plateauing at high ratios, which differs from previous reports showing that increasing the amount of seeds increases the rate of polymerization proportionally.^8,10^ Indeed, ratios of 1/10 and 1/20 result in almost the same rate of polymerization (Figure 4a). This result suggests that, in addition to the nucleation step, the activation step of the trapped monomeric state also contributes to the overall rate of polymerization. Thus, the overall process is characterized by a significant thermal activation barrier. A kinetic model of the processes involved is described below, based on Zhao and Moore (Figure 5).^21^

We consider two steps to reach the self-assembled state starting from the trapped monomers (Figure 5). The first step consists in the activation of the trapped monomeric state M, which involves breaking of all of the hydrogen-bonded rings and the conformational reorientation of the molecule. The second step involves the collision and the successful binding of the activated monomer to the growing end of the seed. The concentration and lifetime of the molecules in the activated monomeric state ([M*]) is considerably lower than the concentration and lifetime of the molecules in the trapped monomeric state ([M]) and as part of the seed ([S]) throughout the whole process. Consequently, it is reasonable to assume that the concentration changes of the activated monomeric state are negligible during the whole process. Equation 1 then describes this assumption during the overall self-assembly process from the trapped monomeric state to the assembled state
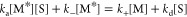
1where *k*_+_ is the rate constant of the transition from the trapped monomeric state to the activated monomeric state, *k*_–_ is the rate constant of the transition from the activated monomeric state to the trapped monomeric state, *k*_a_ is the addition rate constant of an activated monomer to the growing end of a seed, and *k*_d_ is the dissociation rate constant of an activated monomer from the seed. Based on the model in Figure 5, the conversion rate of the trapped monomeric state can be described by eq 2
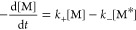
2Combining eqs 1 and 2 leads to eq 3, which expresses the rate of conversion of the trapped monomeric state with no dependence on the concentration of activated monomers ([M*])
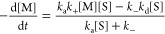
3When the concentration of seeds is low, *k*_–_ ≫ *k*_a_[S], and eq 3 can be approximated as eq 4
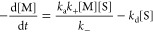
4

If the rate-determining step was the collision between the activated monomer and the growing end of the seed, the rate of polymerization would be proportional to [S], as described in previous reports.^8,10^ In our case, the rate-determining step is the thermal activation of the trapped monomeric state because the rate of polymerization becomes independent to [S] at high concentrations of seeds. Consequently, when the number of active seed growing ends is high, *k*_a_ [S] ≫ *k*_–_Eq 3 can be approximated as eq 5
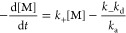
5

**Figure 6:**
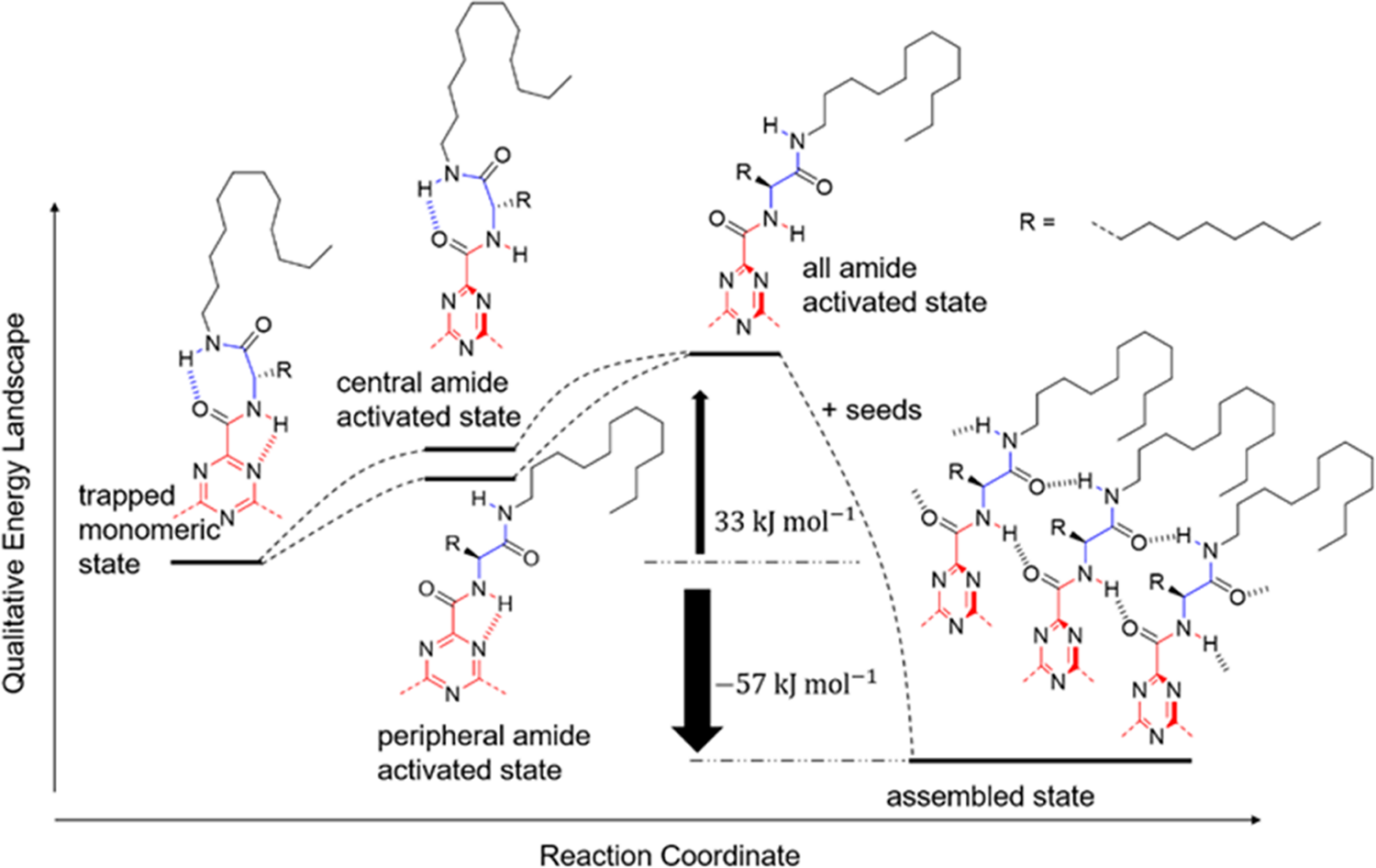
Energy landscape of the polymerization of a single arm of TTA 4. TTA 4 can only polymerize when all three arms are activated. The kinetic barrier of the whole molecule in the self-assembly process is Δ*E*^#^ = 98 kJ mol^–1^, and the elongation enthalpy of the whole molecule is Δ*H*_e_ = −171 kJ mol^–1^. The Kinetic barrier of each arm is Δ*E*_arm_^#^ = Δ*E*^#^/3 = 33 kJ mol^–1^. The elongation enthalpy contribution of each arm is Δ*H*_e arm_ = Δ*H*_e_/3 = −57kJ mol^–1^.

Equation 5 was used to fit the evolution of the CD signal during the seeding experiments with the ratios of 1/10 and 1/20 (Figure S11) at 298 and 288 K, respectively. The value of *k*_+_ does not increase significantly for higher seed ratios for both temperatures (Figure S11). This means that, at the ratio of 1/10, the condition matched the boundary conditions of eq 5. *k*_+_ was determined at four different temperatures (298, 288, 283, and 278 K) by seeding at the ratio of 1/10 (Figure 4b). The Arrhenius plot (Figure 4c) gives the height of the kinetic barrier Δ*E*^#^ = 98 kJ mol^–1^. When crossing this kinetic barrier, each arm of TTA 4 has to break its two intramolecular hydrogen bonds at the same time. So, the kinetic barrier for each arm is Δ*E*_arm_^#^ = Δ*E*^#^/3 = 33 kJ mol^–1^ (Figure 6). Following this, the thermodynamic model proposed by Meijer at al.^22^ was fitted to the TTA 4 heating curve (Figure S13d) to get the elongation enthalpy Δ*H*_e_ = −171 kJ mol^–1^. The contribution of each arm is Δ*H*_e,arm_ = Δ*H*_e_/3 = −57 kJ mol^–1^. The single-arm energy landscape of the self-assembly of TTA 4 is schematically summarized in Figure 6.

### Hetero-Seeding

**Figure 7:**
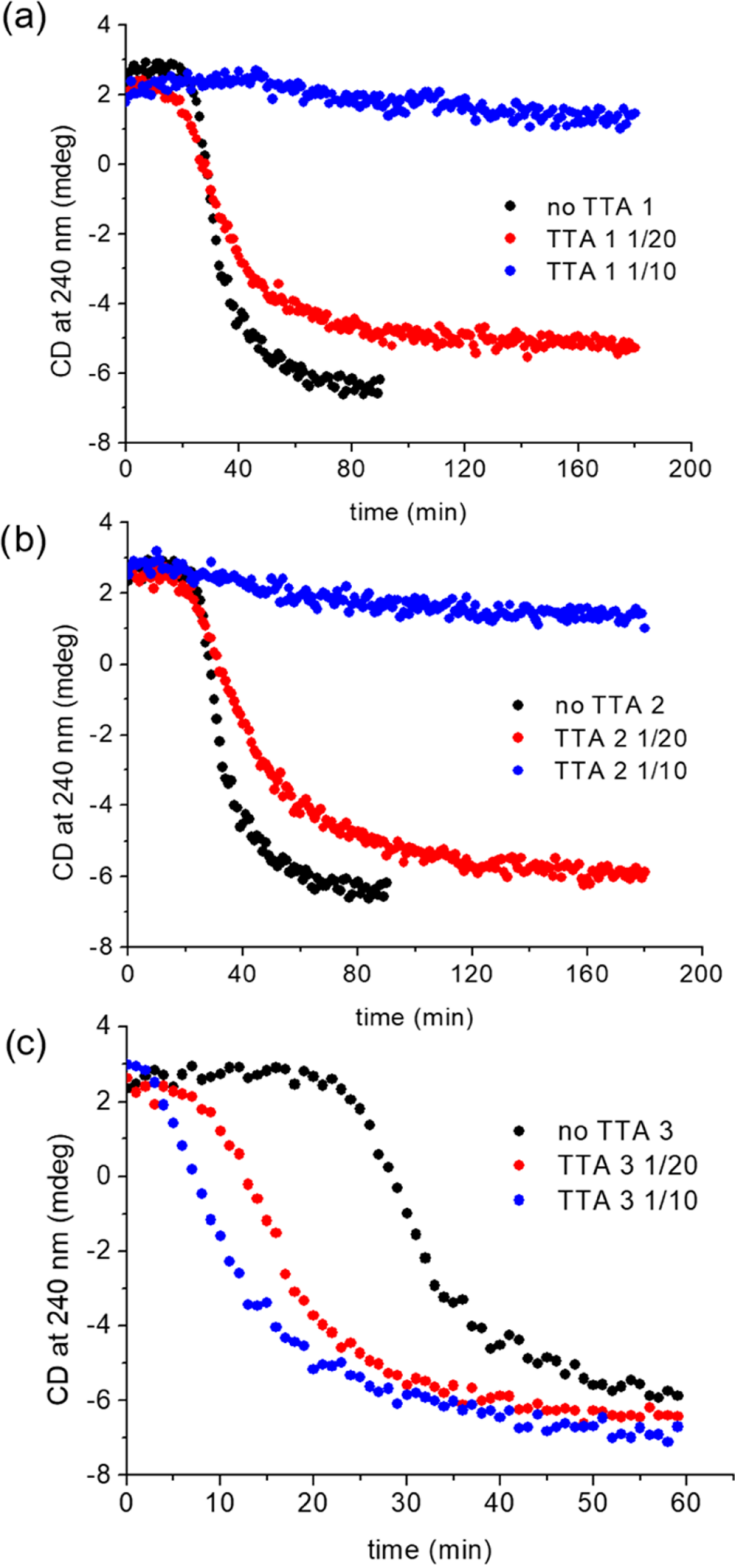
Hetero-seeding of various ratio of (a) TTA 1, (b) TTA 2, and (c) TTA 3 to a solution of 30 μM TTA 4 at 298 K after cooling from 358 K at the rate of 10 K min^–1^.

Further, we investigated the influence of the addition of seeds of TTA 1, TTA 2, and TTA 3 on the polymerization of TTA 4. All seeds were prepared from 2 mM stock solutions of TTA 1–3 by 20 s of sonication. The influence of sonication was investigated by UV–vis (Figure S9a–c), CD (Figure S9e–g), and TEM (Figure S10a–c). Seeds of TTA 1 and TTA 2 significantly slow the polymerization of TTA 4 at the ratio of 1/20 and almost fully inhibit the assembly process at the ratio of 1/10 (Figure 7a,b).

The origin of this inhibition most likely stems from the similarity of the core that allows the binding of TTA 4 to the seeds of TTA 1 and TTA 2. However, the fine differences in the sterics of the side groups render the conformation of the bound TTA 4 incapable of promoting further growth. Contrary to the inhibition of the self-assembly of TTA 4 by the seeds of TTA 1 and 2, the seeds of TTA 3 significantly enhance the rate of TTA 4 polymerization (Figure 7c). This is attributed to the greater similarity of the conformation of the self-assembled states of TTA 3 and 4. The activated monomer of TTA 4 bound to the seed made of TTA 3 provides a growing end that effectively binds another activated monomer of TTA 4 and promotes the growth of the TTA 4 fiber. The hetero-seeding process was also investigated by DLS. These investigations confirm that TTA 3 seeds can effectively trigger the polymerization of TTA 4 monomers (Figure S12). TTA 1 and TTA 2 seeds did not accelerate the polymerization of TTA 4 monomers, which is consistent with their inhibitory effect on TTA 4 polymerization discussed above (Figure S12).

## Conclusions

Seeded supramolecular polymerization has developed into a relevant strategy to engineer the properties and function of supramolecular materials beyond the molecular design.^4^ This approach has already been applied to the synthesis of PN junction-based nanodevices.^23^ However, designing monomers with appropriate kinetic barriers remains the limiting factor. The reported strategy to overcome this limitation presented here relies on combining two interdependent hydrogen-bonding motives that can undergo the transition from intramolecularly bound state to the intermolecularly bound state. Our findings suggest that an additional control of steric interactions between the monomers can further tune the kinetic barriers and influence the nucleation time. A kinetic model of the self-assembly process including the thermal activation of the monomer was proposed, and the activation barrier was determined by the Arrhenius plot. When the seed concentration is high enough, the thermal activation of the monomeric state becomes the rate-determining step. The polymerization of TTA 4 triggered by seeds of TTA 1, TTA 2, and TTA 3 was performed. It was found that the difference between the steric interactions due to the length of the side groups results either in acceleration or inhibition of the self-assembly process. Presented findings might be relevant for designing systems featuring seeded supramolecular polymerization and more generally supramolecular materials with greater control of the polymerization processes.^14^

## Supporting Information Available

All experimental details; synthetic procedure; CD, UV−vis, FTIR, TEM, AFM data; DLS data; ^1^H NMR, ^13^C NMR, and ESI-MS data (PDF)
