# Supplementary material for: Molecular Engineering of the Kinetic Barrier in Seeded Supramolecular Polymerization
Source: J Am Chem Soc. 2023 Feb 24;145(9):5053–60. doi: 10.1021/jacs.2c10482 (PMC9999411; doi:10.1021/jacs.2c10482)
Supplement: Supplementary file 1 — ja2c10482_si_001.pdf [file ja2c10482_si_001.pdf]

# Molecular engineering of the kinetic barrier in seeded supramolecular polymerization

Qin Huang,† Nicolas Cissé,† Marc C. A. Stuart,‡ Yaroslava Lopatina,† and Tibor Kudernac*†.

† Stratingh Institute for Chemistry, University of Groningen, Nijenborgh 4, 9747 AG Groningen (The Netherlands).

‡ Groningen Biomolecular Sciences and Biotechnology Institute, University of Groningen, Nijenborgh 7, 9747 AG Groningen (The Netherlands).

*To whom correspondence should be addressed

E-mail: t.kudernac@rug.nl

**Table of Content**

**1. Materials and Methods** .......... 3

**2. $^1$H NMR and CD spectra of TTA 0 in methylcyclohexane-d$_{14}$ (MCH-d$_{14}$)** .......... 4

**3. Investigation of the intramolecular H-bonding by IR spectroscopy** .......... 5

**4. Self-assembly mechanism of TTA 1 - TTA 2.** .......... 7

**5. Heating and cooling of TTA 1 – TTA 3 solutions followed by CD spectroscopy and TEM investigations at room temperature.** .......... 8

**6. AFM microscopy of TTA 1 - TTA 4** .......... 9

**7. Heating and cooling of TTA 1 - TTA 4 solutions followed by UV-Vis spectroscopy** .......... 10

**8. Heating and cooling of TTA 3 and TTA 4 solutions at a lower concentration followed by CD spectroscopy** .......... 11

**9. Time-resolved supramolecular polymerization of TTA 3-4 followed UV-Vis spectroscopy** .......... 14

**10. Investigation of sonication influence on TTA 1 - TTA 4 by CD and UV-Vis** .......... 15

**11. Investigation of sonication influence on TTA 1 - TTA 4 by TEM** .......... 16

**12. Seeded supramolecular polymerization of TTA 4 followed by CD spectroscopy.** .......... 17

**13. Seeded and hetero-seeded supramolecular polymerization of TTA 4 followed DLS** .......... 18

**14. Heating curve of TTA 1 - TTA 4 fitted to a cooperative model.** .......... 20

**15. TEM investigation of morphology TTA 4 morphology triggered by TTA 3 seeds and TTA 4 seeds** .......... 21

**16. Temperature-dependent $^1$H NMR spectroscopy of TTA 4.** .......... 22

**17. Synthesis** .......... 23

**18. Characterization** .......... 27

**19. References** .......... 41

## 1. Materials and Methods

**Materials:** All Solvents and reagents were received from Acros, Aldrich TCI or Merck and were used without further purification, unless otherwise mentioned. The Benzene-1, 3, 5-tricarboxamides (BTA) model compound was synthesized according to a protocol found in literature.[1]

**Methods:** $^{1}$H NMR and $^{13}$C NMR spectra were obtained on a Varian AMX400 ($^{1}$H: 400 MHz, $^{13}$C: 101 MHz) spectrometer at room temperature (25 °C). Temperature dependent $^{1}$H NMR for self-assembly characterization were obtained on a Varian Unity Plus ($^{1}$H: 500 MHz, $^{13}$C: 125 MHz) spectrometer. Mass spectra (MS) were obtained on a Waters Xevo G2 TOF spectrometer with ESI ionization. Flash column chromatography was performed on BUCHI Pure C-810 Flash system with commercial column Flash Pure ID Silica 40μm (12 g or 40 g). The UV-Vis absorption spectra were obtained on an Agilent Technologies Cary 8454 UV-Vis spectrometer with 1 cm path length quartz cuvette. The circular dichroism (CD) absorption spectra were obtained on a JASCO-815 CD spectrometer with 1 cm path length quartz cuvette. The liquid-phase IR spectra were obtained at room temperature (25 °C) on a Perkin-Elmer FT-IR Spectrometer 400 with a demountable liquid cell (KBr windows and PTFE spacer 1mm path length). TEM images were taken with a CM120 microscope at 120keV acceleration voltage on plain carbon coated grids. The atomic force microscopy (AFM) imaging was performed by a PicoLE microscope (Molecular imaging) using ACAFM mode. Standard silicon nitride cantilevers (PointProbe, Nanosensors) with resonance frequency 320 kHz were used. Muscovite mica sheets (EMS) were used as a substrate. AFM samples were prepared by drop casting. DLS measurements were performed at room temperature on a Malvern Panalytical Zetasizer Ultra Red equipped with a 1 cm quartz cuvette.

Seeds preparation: All seeds are prepared by sonicating 2 mM assembled TTA 1 - TTA 4 solution for 20 s at r.t. Before the addition of TTA 4 seeds, extra MCH is used to dilute the seeds to 30 μM and add to the 30 μM TTA 4 monomer solution at the selected volume ratio. For the addition of TTA 1 - TTA 3 seeds to the 30 μM TTA 4 monomer solution, the sonicated 2 mM are directly added to the TTA 4 solution with the selected volume assuming the total volume of solution after addition is unchanged.

## 2. $^{1}H$ NMR and CD spectra of TTA 0 in methylcyclohexane-$d_{14}$ (MCH-$d_{14}$).

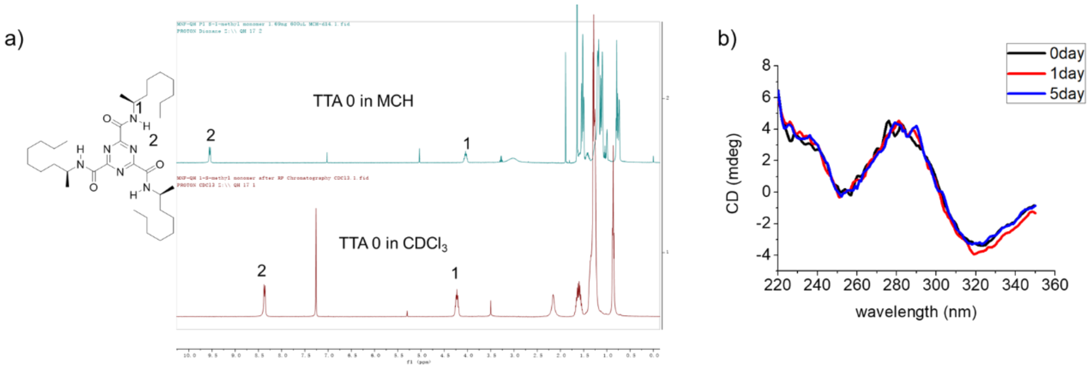


**Figure S1.** (a) $^{1}H$ NMR spectrum of TTA 0 in MCH-$d_{14}$ at 4.78 mM after fast cooling from MCH boiling point to room temperature and $^{1}H$ NMR spectrum of TTA 0 that is molecularly dissolved in $CDCl_3$. (b) CD spectrum of TTA 0 dissolved at 200 μM in MCH, freshly dissolved, and after 1 and 5 days of ageing at room temperature.

## 3. Investigation of the intramolecular H-bonding by IR spectroscopy

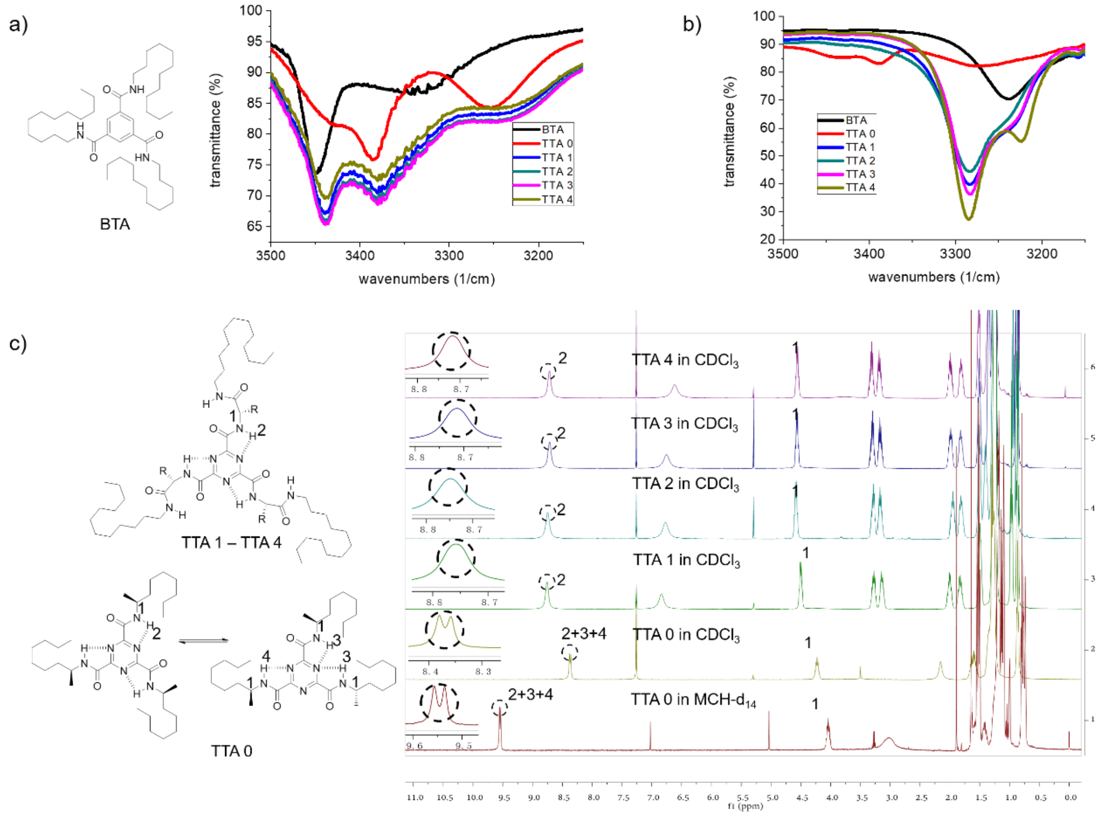


**Figure S2.** (a) IR spectra of all compounds at 2 mM in $CHCl_3$ and molecular structure of the BTA model compound used as a control. (b) IR spectra of all compounds at 2 mM in MCH. c) $^1H$ NMR spectrum of TTA 0 in MCH-$d_{14}$ and $^1H$ NMR spectrum of TTA 0 - TTA 4 in $CDCl_3$.

First, we investigate the IR spectra of all compounds in $CHCl_3$ (Figure S2a). The infrared spectrum of TTA 0 in $CHCl_3$ reveals a N-H stretch centered at 3386 $cm^{-1}$ with a shoulder at 3434 $cm^{-1}$ , corresponding to the intramolecularly hydrogen bonded protons 2 and proton 4 respectively (see proton attribution on Figure S2c TTA 0). The N-H stretch centered at 3256 $cm^{-1}$ corresponds to protons 3 (Figure S2c TTA 0 right conformer). This mode of hydrogen bonding is also supported by the $^1H$ NMR spectroscopy of TTA 0 in $CDCl_3$ (Figure S2c), revealing a dedoubling of the amide peak, corresponding to the intramolecular hydrogen bonds formed between one nitrogen atom and one hydrogen atom (protons 2 and 4) and the intramolecular hydrogen bonds formed between one nitrogen atom and two hydrogen atoms (protons 3).

The IR spectrum of the BTA control building block, which cannot form any intramolecular hydrogen bonds, reveals a N-H stretch centered at 3448 $cm^{-1}$ in $CHCl_3$ (non-hydrogen-bonded state). This wavenumber is higher than all three N-H stretches of TTA 0, such shift indicates the difference between intramolecularly hydrogen-bonded states and non-hydrogen-bonded states.[2]

Like TTA-0, the IR spectra of TTA 1 - TTA 4 in $CHCl_3$ (Figure S2a) indicates intramolecular H-bonding, as revealed by the N-H stretch centered around 3380 $cm^{-1}$, attributed to the central amide protons 2 (Figure S2c TTA 1 - TTA 4). The difference with TTA 0 is that there is no N-H stretch centered at 3256 $cm^{-1}$ (protons 3) but rather a shoulder. This is probably because of the longer side chains of TTA 1 - TTA 4. This steric effect prevents the formation of the intramolecular hydrogen bonds between two hydrogen atoms and one nitrogen atom (protons 3). $^1H$ NMR spectrum of TTA 1 - TTA 4 in $CDCl_3$ (Figure S2c) also

matches this assumption. Indeed, there is no dedoubling of the amide peak for TTA 1 - TTA 4 in $CDCl_3$ (only a broad singlet i.e protons 2).

We attribute the N-H stretch centered at 3439 $cm^{-1}$ to the peripheral amide groups of TTA 1 - TTA 4. Unlike TTA 0, there is no shoulder at 3434 $cm^{-1}$ (proton 4) because in TTA 1 – TTA 4, this potential band is covered by the intense N-H stretches of the peripheral amide groups and therefore it is difficult to investigate this aspect by IR spectroscopy. It is also difficult to conclude on the formation of intramolecular hydrogen bonds by the peripheral amide groups of TTA 1 - TTA 4 in chloroform, even though the absence of intermolecular hydrogen bonds in chloroform is as obvious as in the BTA control, as discussed bellow.

The IR spectra of all compounds in MCH is shown in Figure S2b. The IR spectrum of the BTA control in MCH reveals an N-H stretch centered at 3241 $cm^{-1}$, this large shift (3448 $cm^{-1}$ in $CHCl_3$) towards lower wavenumbers, indicates the typical intermolecular hydrogen bonding of BTA fibers in MCH.

The infrared spectrum of TTA 0 in MCH (Figure S2b) is similar to its spectrum in $CHCl_3$. It reveals an N-H stretch centered at 3391 $cm^{-1}$ (corresponding to the N-H stretch centered at 3386 $cm^{-1}$ in $CHCl_3$) with a shoulder at 3437 $cm^{-1}$ (corresponding to the shoulder at 3434 $cm^{-1}$ in $CHCl_3$). It also reveals an N-H stretch centered at 3270 $cm^{-1}$ (corresponding to the N-H stretch centered at 3256 $cm^{-1}$ in $CHCl_3$). And similarly to its $^1H$ NMR spectrum in $CDCl_3$, the $^1H$ NMR spectrum of TTA 0 in MCH-$d_{14}$ also reaveals a dedoubling of the amide peaks (Figure S2c). So these results suggest that TTA 0 is intramolecularly hydrogen bonded in chloroform as well as in MCH (TTA 0 monomers).

On the contrary, the IR spectra of TTA 1 - TTA 4 in MCH reveals a large shifts of the N-H stretches towards lower wavenumber in comparison to their N-H stretches in chloroform (Figure S2b). The N-H stretches attributed to the peripheral amide groups decrease from 3439 $cm^{-1}$ to 3286 $cm^{-1}$. And the N-H stretches attributed to the central amide groups decrease to wavenumber close to those of the BTA fibers. For TTA 1 – TTA 3, they decrease from 3380 $cm^{-1}$ to 3235 $cm^{-1}$ (into a shoulder), while for TTA 4 it decreases from 3380 $cm^{-1}$ to 3225 $cm^{-1}$ (into a relatively sharper peak). The large shifts towards lower wavenumbers of both central amide groups and peripheral amide groups indicate that these two types of amide groups are intermolecularly hydrogen bonded in MCH (TTA 1 – TTA 4 fibers).

## 4. Self-assembly mechanism of TTA 1 - TTA 2.

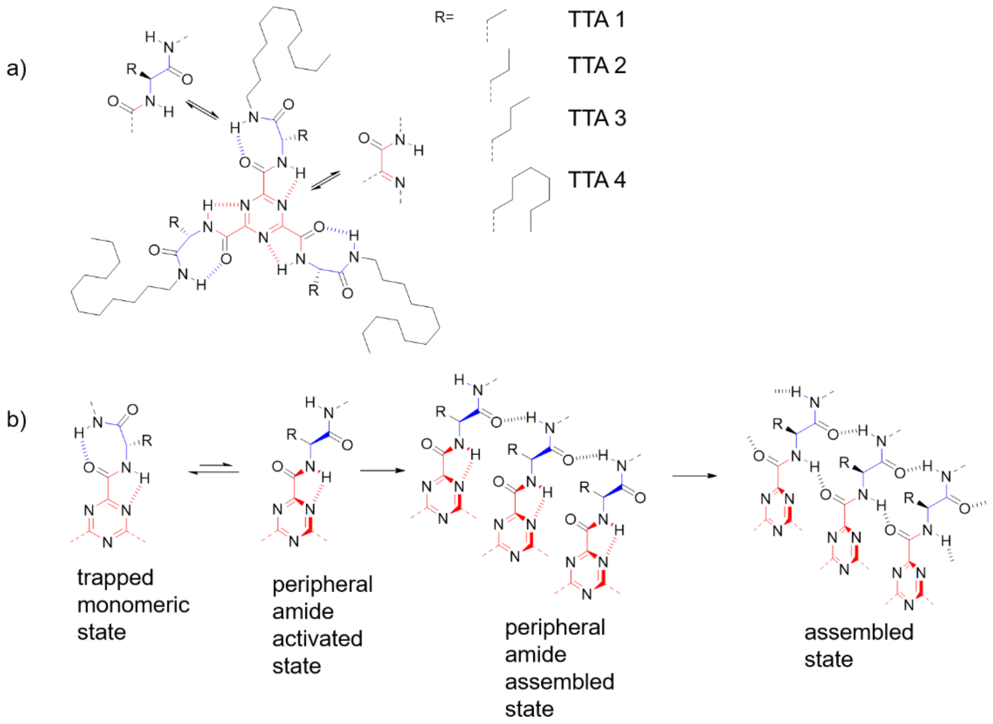


**Figure S3.** (a) Molecular structure and conformations of TTA 1 - TTA 4. The central amide group and the peripheral amide groups are represented in their intramolecularly-bonded state and their non-intramolecularly-bonded state. (b) Self-assembly process of TTA 1 - TTA 2.

If R is not bulky (TTA 1, TTA 2) breaking solely the peripheral hydrogen bonding is sufficient to trigger polymerization. Indeed, when the three peripheral amides are activated while the central amides maintain their intramolecular hydrogen bonding, the molecule can self-assemble into the peripheral amide assembled state. So the free energy of intermolecular hydrogen bonding of the central amide groups decreases (less entropic penalty) in comparison to the free energy of intermolecular hydrogen bonding of the central amide groups directly from the monomeric state. Because the free energy of bonding has decreased, it can be below 0, then the central amide groups can switch intramolecular hydrogen bonding to intermolecular hydrogen bonding while keeping the peripheral amide intermolecular bonded. The intermolecular hydrogen bonding of both central and peripheral amides is also supported by the IR spectra in MCH (Figure S2b). However, this detail didn't change the unsuccessful synchronized activation of the central and peripheral amides for TTA 1 and TTA 2.

**5. Heating and cooling of TTA 1 – TTA 3 solutions followed by CD spectroscopy and TEM investigations at room temperature.**

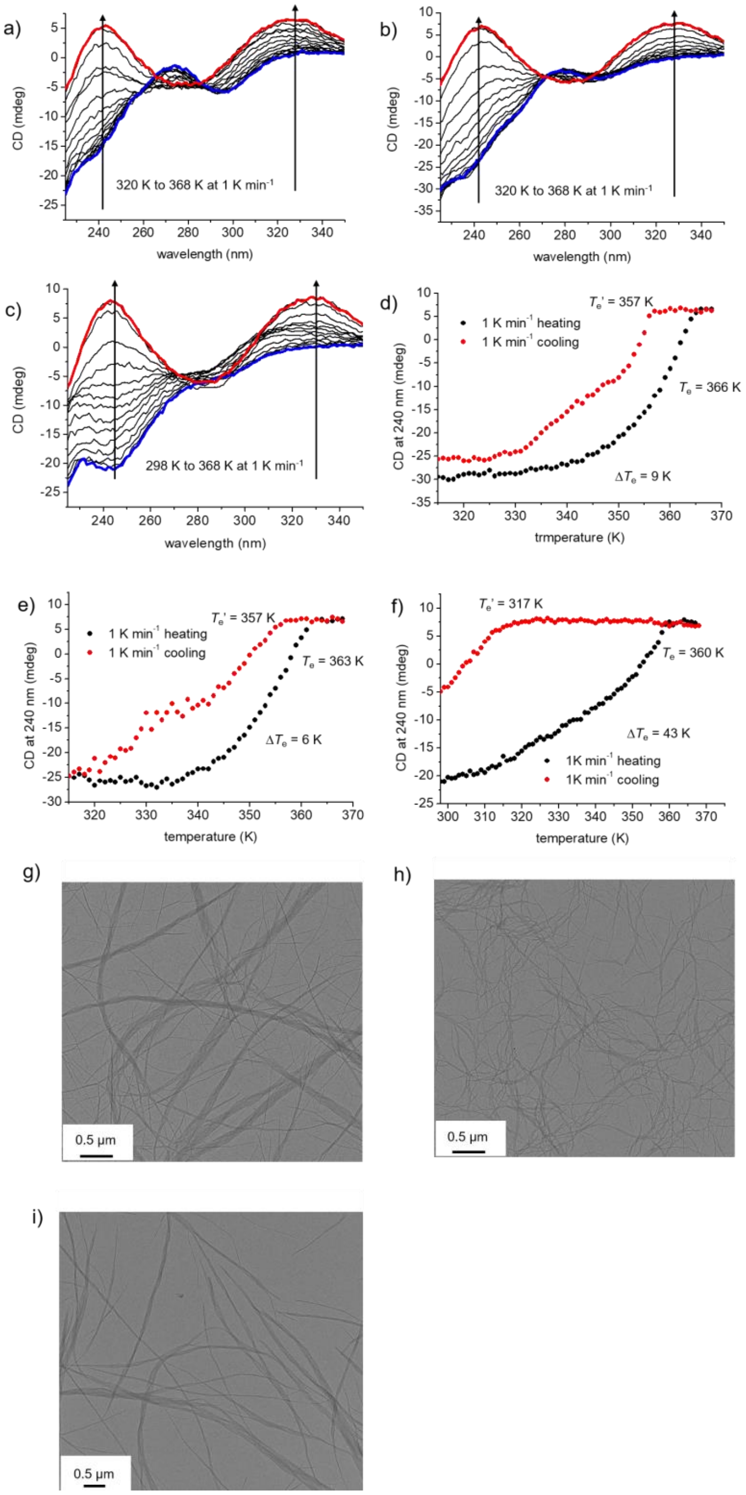


**Figure S4.** All temperature dependent CD spectra are measured in MCH at a building block concentration of 100 μM at a heating/cooling rate of 1 K $min^{-1}$. (a) TTA 1 (b) TTA 2 (c) TTA 3 (d) CD at 240 nm for TTA 1 (e) CD at 240 nm for TTA 2 (f) CD at 240 nm for TTA 3 (g) TEM micrograph of TTA 1 at 100 μM assembled in MCH at 298 K and dried. (h) TEM micrograph of TTA 2 at 100 μM assembled in MCH at 298 K and dried. (i) TEM of TTA 3 at 100 μM assembled in MCH at 298 K and dried.

## 6. AFM microscopy of TTA 1 - TTA 4

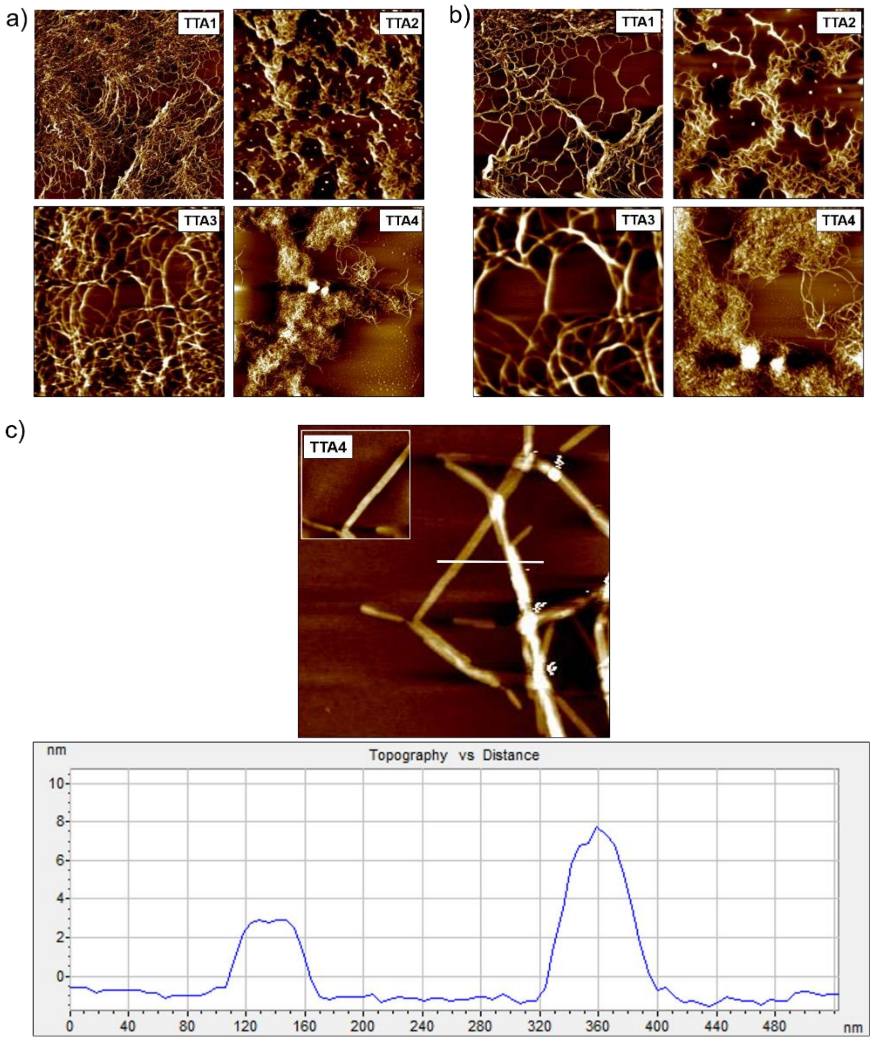


**Figure S5**. AFM micrograph of TTA 1 - TTA 4 at 100 μM assembled in MCH at 298 K and dried. The size of each image is (a) 90*90 μm² (b) 40*40 μm² (c) 1.25*1.25 μm² (inset: 0.5*0.5 μm²). (c) bottom: hight profiles along the white line.

AFM images of TTA 1 - TTA 4 at a larger (Figure S5a, b) scale show a fiber-like morphology.

Reduced-scale images allow visualizing thinner fibers, such as for TTA 4 in Figure S5c. The inhomogeneity of the contrast along a fiber can be explained by the presence of long flexible alkyl chains.

We assume that alkyl chains prevent determining the structure of the packing of the aromatic core of TTA 1 - TTA 4 due to the insufficient stiffness and density of the packing to reproduce the internal structure of the fiber.

## 7. Heating and cooling of TTA 1 - TTA 4 solutions followed by UV-Vis spectroscopy

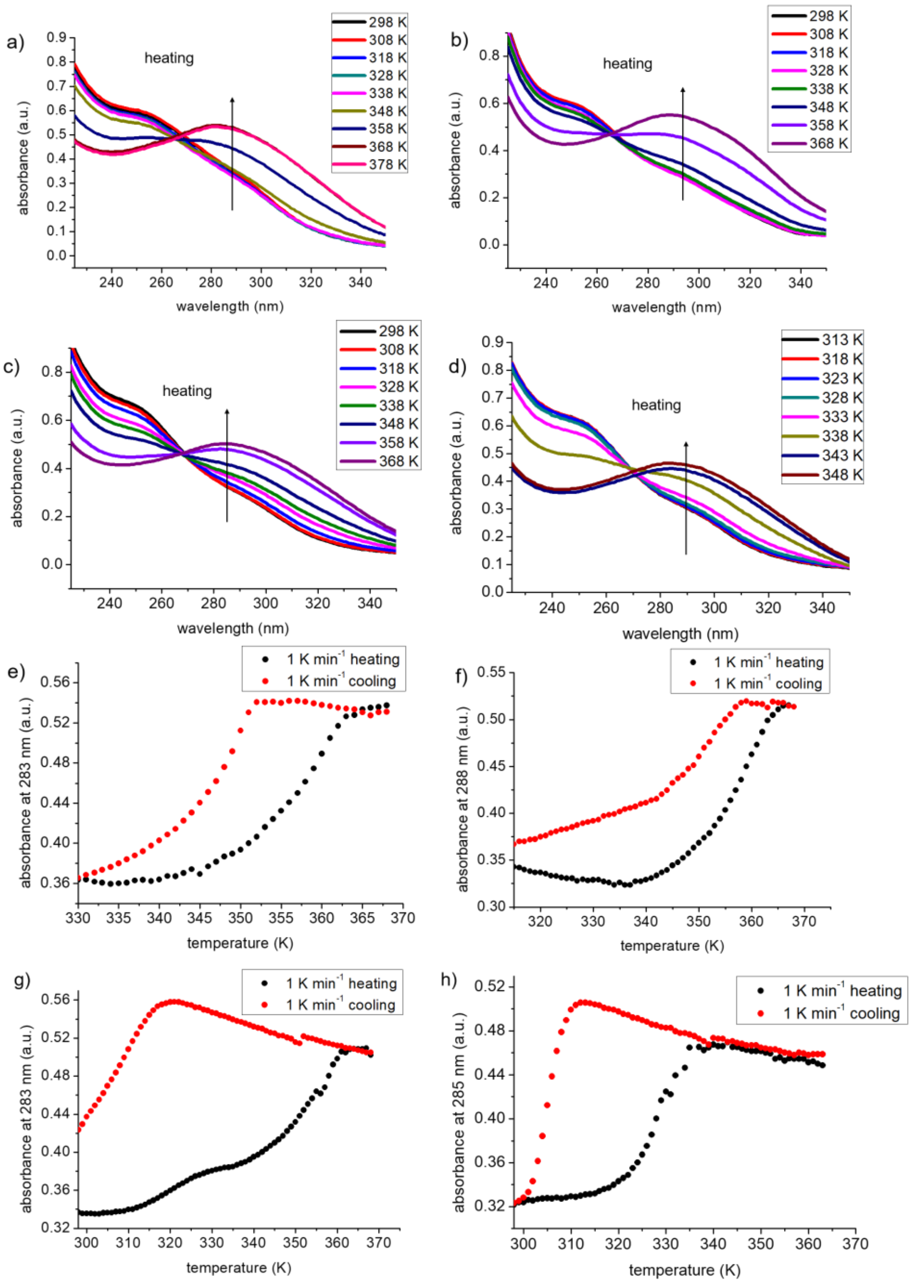


**Figure S6.** All UV-Vis spectra are measured in MCH at a building block concentration of 100 μM, the heating rate and the cooling rate is 1 K min$^{-1}$. (a) UV-Vis spectrum of TTA 1 (b) UV-Vis spectrum of TTA 2 (c) UV-Vis spectrum of TTA 3 (d) UV-Vis spectrum of TTA 4. (e) Absorbance of TTA 1 at 283 nm as a function of temperature (f) Absorbance of TTA 2 at 288 nm as a function of temperature. (g) Absorbance of TTA 3 at 283 nm as a function of temperature . (h) Absorbance of TTA 4 at 285 nm as a function of temperature .

## 8. Heating and cooling of TTA 3 and TTA 4 solutions at a lower concentration followed by CD spectroscopy

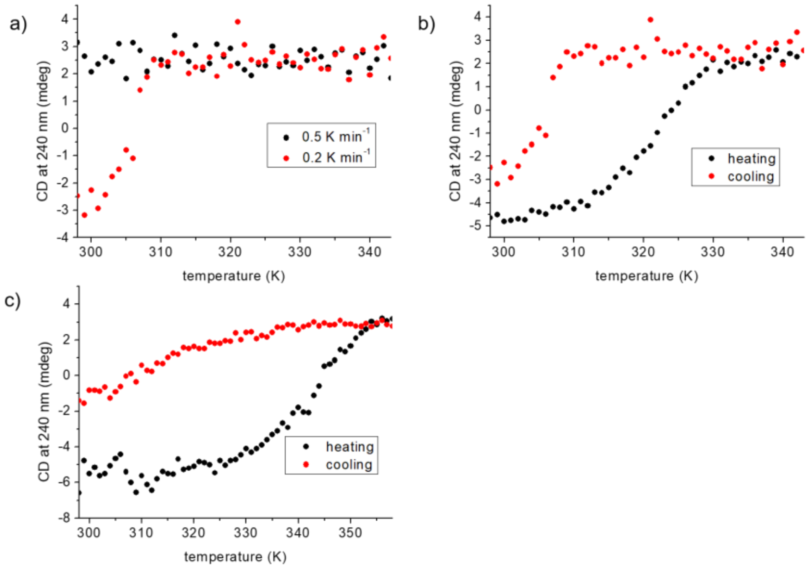


**Figure S7.** (a) CD at 240 nm of TTA 4 at 30 μM in MCH upon cooling at 0.5 K min$^{-1}$ and 0.2 K min$^{-1}$. (b) CD at 240 nm of TTA 4 at 30 μM in MCH upon heating and cooling at 0.2 K min$^{-1}$. Critical elongation temperature upon heating ($T_e$) is 330 K and upon cooling ($T_e{}'$) is 309 K. The $\Delta T_e = T_e - T_e{}'$ is 21 K. (c) CD at 240 nm of TTA 3 at 30 μM in MCH upon heating and cooling at 0.2 K min$^{-1}$. The $T_e$ is 352 K and the $T_e{}'$ is 343 K. The $\Delta T_e$ is 9 K. All critical elongation temperatures are obtained by fitting the curve to the model proposed by Meijer, Schenning et al.[3]

Here we investigate the kinetic barrier again by thermal hysteresis at a lower concentration to compare the kinetic barrier between TTA 4 and TTA 3. As Figure S7 shows, the $\Delta T_e$ of TTA 4 is 21 K. This is much higher than the $\Delta T_e$ of TTA 3 which is only 9 K.

This is because at the lower concentration influence of fiber fragmentation on nucleation time is reduced and the main contribution comes from the primary nucleation which reflects the height of the real kinetic barrier. As the hysteresis comes from the nucleation time, TTA 4 which has a higher kinetic barrier shows a bigger $\Delta T_e$.

For more details, the reduction of influence from fiber fragmentation on nucleation time at lower concentrations can be explained below.

During the nucleation phase, the process is proposed as follows:

$$n_c \mathrm{M}^* \xrightarrow{k_n^*} \mathrm{S}_{n_c} \quad \text{(eq S1)}$$

$$\mathrm{S}_i + \mathrm{M}^* \xrightarrow{k_{ma}^*} \mathrm{S}_{i+1} \quad \text{(eq S2)}$$

$$\mathrm{S}_{i+j} \xrightarrow{k_f} \mathrm{S}_i + \mathrm{S}_j \quad \text{(eq S3)}$$

Where $\mathrm{M}^*$ is the activated monomer, $\mathrm{S}_i$ is the fiber with length $i$, $n_c$ is the size of the nucleus, $k_n^*$ is the nucleation rate constant, the nucleation, $k_{ma}^*$ is the elongation rate constant, and $k_f$ is the fragmentation rate constant. Here we ignore the reversibility. Because during the nucleation phase, the rate of associating $\mathrm{M}^*$ to $\mathrm{S}_i$ is very low due to the low concentration of $\mathrm{S}_i$, we can also assume $[\mathrm{M}^*] =$

$\alpha$[M]. Where [M*] is the concentration of M* and [M] is the concentration of trapped monomer M and [M*] ≪ [M]. $\alpha$ is a constant which is concentration-independent. Therefore we can replace M* with M in the eq S1 and eq S2.

Then the reduction of the influence of fragmentation at lower concentrations can be explained by the model proposed by Kym Eden et al.[4] They assume primary nucleation (eq S4), fibril elongation (eq S5), and fragmentation (eq S6) happens during the self-assembly process. Because replacing M* with M doesn't change the equation deduced by fragmentation, eq S6 is unchanged compared with eq S3.

$$n_{\mathrm{c}}\mathrm{M} \xrightarrow{k_{\mathrm{n}}} \mathrm{S}_{n_{\mathrm{c}}} \qquad \text{(eq S4)}$$

$$\mathrm{S}_i + \mathrm{M} \xrightarrow{k_{\mathrm{ma}}} \mathrm{S}_{i+1} \qquad \text{(eq S5)}$$

$$\mathrm{S}_{i+j} \xrightarrow{k_{\mathrm{f}}} \mathrm{S}_i + \mathrm{S}_j \qquad \text{(eq S6)}$$

Where M is the trapped monomer, $\mathrm{S}_i$ is the fiber with length $i$, $n_{\mathrm{c}}$ is the size of the nucleus, $k_{\mathrm{n}} = \alpha^{n_{\mathrm{c}}} k_{\mathrm{n}}^*$ is the nucleation rate constant, the nucleation, $k_{\mathrm{ma}} = \alpha k_{\mathrm{ma}}^*$ is the elongation rate constant and $k_{\mathrm{f}}$ is the fragmentation rate constant which is the same as the one in eq S3.

With this, they proposed an equation to calculate the nucleation time:

$$\tau_{\mathrm{n}} = (1/\kappa)\ln\left(1 - \Phi D^{-1} + ((\Phi D^{-1})^2 - 2\Phi D^{-1})^{1/2}\right) + n_{\mathrm{c}}!/\left(V N_{\mathrm{A}} k_{\mathrm{n}} [\mathrm{M}]_{\mathrm{tot}}^{n_{\mathrm{c}}}\right) \qquad \text{(eq S7)}$$

$\tau_{\mathrm{n}}$ is the nucleation time which is defined as the time that a certain ratio r of molecules transforms into the assembled state (in their research the ratio r is 3%). $[\mathrm{M}]_{\mathrm{tot}}$ is the total concentration of molecules including the monomers and the molecules in the assembled state. $\kappa = (2k_{\mathrm{ma}}k_{\mathrm{f}}[\mathrm{M}]_{\mathrm{tot}})^{1/2}$, $\Phi = \ln(1-r)$, $D = \left(k_{\mathrm{n}}[\mathrm{M}]_{\mathrm{tot}}^{n_{\mathrm{c}}-1}\right)/(n_{\mathrm{c}}!\, k_{\mathrm{f}})$, $V$ is the volume of the solution, $N_{\mathrm{A}}$ is the Avogadro constant.

The first term of eq S7 is based on a continuous model to describe the nucleation time with fragmentation. Because the continuous model is not applicable at the early stage when the nucleus amount in the whole solution is less than one due to the discrete nature of primary nucleation, the second term is added. It shows the time cost to form the first nucleus in solution by primary nucleation. While the authors mainly focus on the influence of $k_{\mathrm{n}}$ on $\tau_{\mathrm{n}}$ in their research, here we can use this equation to qualitatively discuss the influence of the $[\mathrm{M}]_{\mathrm{tot}}$ on the contribution of fragmentation to $\tau_{\mathrm{n}}$, especially at low $[\mathrm{M}]_{\mathrm{tot}}$.

Considering the logarithmic term in eq S7 we can get:

$$\ln\left(1 - \Phi D^{-1} + ((\Phi D^{-1})^2 - 2\Phi D^{-1})^{1/2}\right) = \ln\left(1 - \Phi D^{-1} + (-\Phi D^{-1})(1 - 2\Phi^{-1}D)^{1/2}\right).$$

After expanding $(1 - 2\Phi^{-1}D)^{1/2}$ into the Maclaurin series in the second order, we get:

$$(1 - 2\Phi^{-1}D)^{1/2} = 1 + (1/2)(-2\Phi^{-1}D) - (1/8)(-2\Phi^{-1}D)^2 + o((-2\Phi^{-1}D)^2).$$

Then the logarithmic term in eq S7 can be written as:

$$-\ln D + \ln(2D - 2\Phi + (1/2)\Phi^{-1}D^2 + (-\Phi)o((-2\Phi^{-1}D)^2)).$$

Given the condition $D \propto [\mathrm{M}]_{\mathrm{tot}}^{n_{\mathrm{c}}-1}$, we found the ratio of the first term of eq S7 to its second term is proportional to:

$$-D^{(n_{\mathrm{c}}-(1/2))/(n_{\mathrm{c}}-1)}\ln D + D^{(n_{\mathrm{c}}-(1/2))/(n_{\mathrm{c}}-1)}\ln(2D - 2\Phi + (1/2)\Phi^{-1}D^2 + (-\Phi)o((-2\Phi^{-1}D)^2)).$$

As $[\mathrm{M}]_{\mathrm{tot}} \to 0$, because $n_{\mathrm{c}} \geq 2$ we have $D \to 0$, both the first and the second terms in the function above approach 0. Thus when $[\mathrm{M}]_{\mathrm{tot}} \to 0$ the contribution from the first term of eq S7 can be ignored. The main contribution of $\tau_{\mathrm{n}}$ is from the second term which doesn't contain $k_{\mathrm{f}}$ that means the nucleation time is mainly contributed by primary nucleation and the influence of fragmentation is

reduced.

## 9. Time-resolved supramolecular polymerization of TTA 3-4 followed UV-Vis spectroscopy

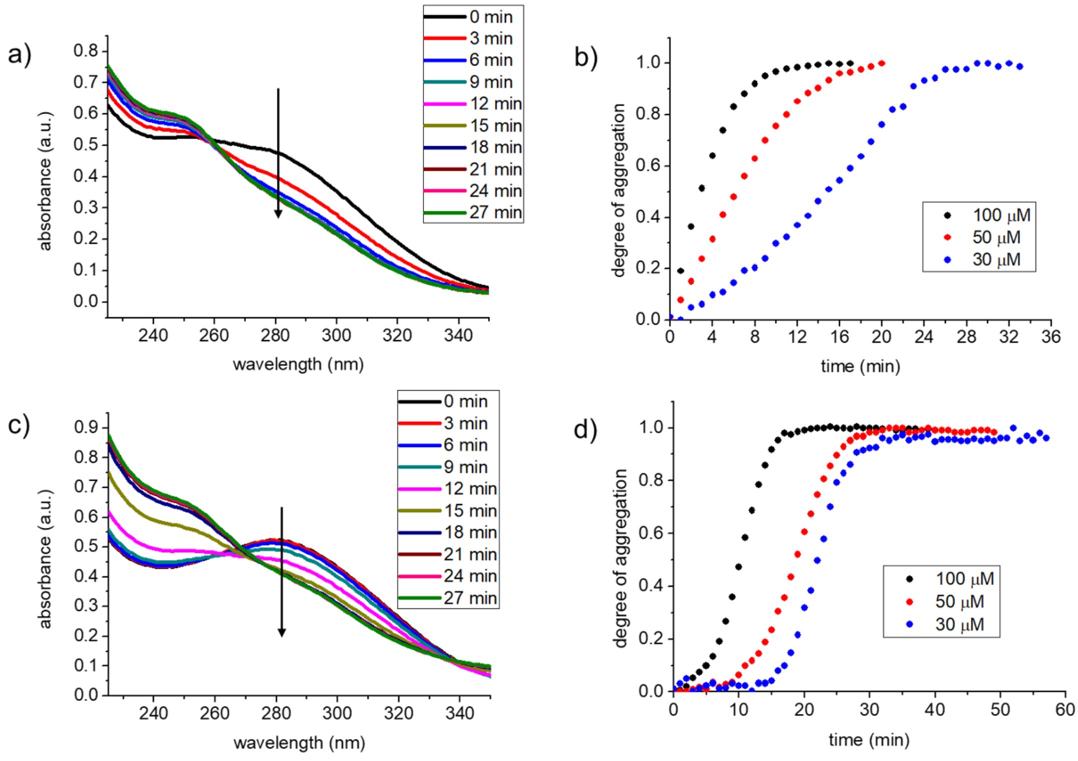


**Figure S8.** (a) UV-Vis spectrum of TTA 3 at 100 μM in MCH after cooling from 368 K to 298 K at a rate of 10 K $min^{-1}$. (b) Degree of aggregation (normalized absorbance at 285 nm) as a function of time for TTA 3 at 100 μM, 50 μM and 30 μM in MCH after cooling from 368 K to 298 K at a rate of 10 K $min^{-1}$ . (c) UV-Vis spectrum of TTA 4 at 100 μM in MCH after cooling from 358 K to 298 K at a rate of 10 K $min^{-1}$. (d) Degree of aggregation (normalized absorbance at 285 nm) as a function of time for TTA 4 at 100 μM, 50 μM and 30 μM in MCH after cooling from 358 K to 298 K at a rate of 10 K $min^{-1}$.

## 10. Investigation of sonication influence on TTA 1 - TTA 4 by CD and UV-Vis

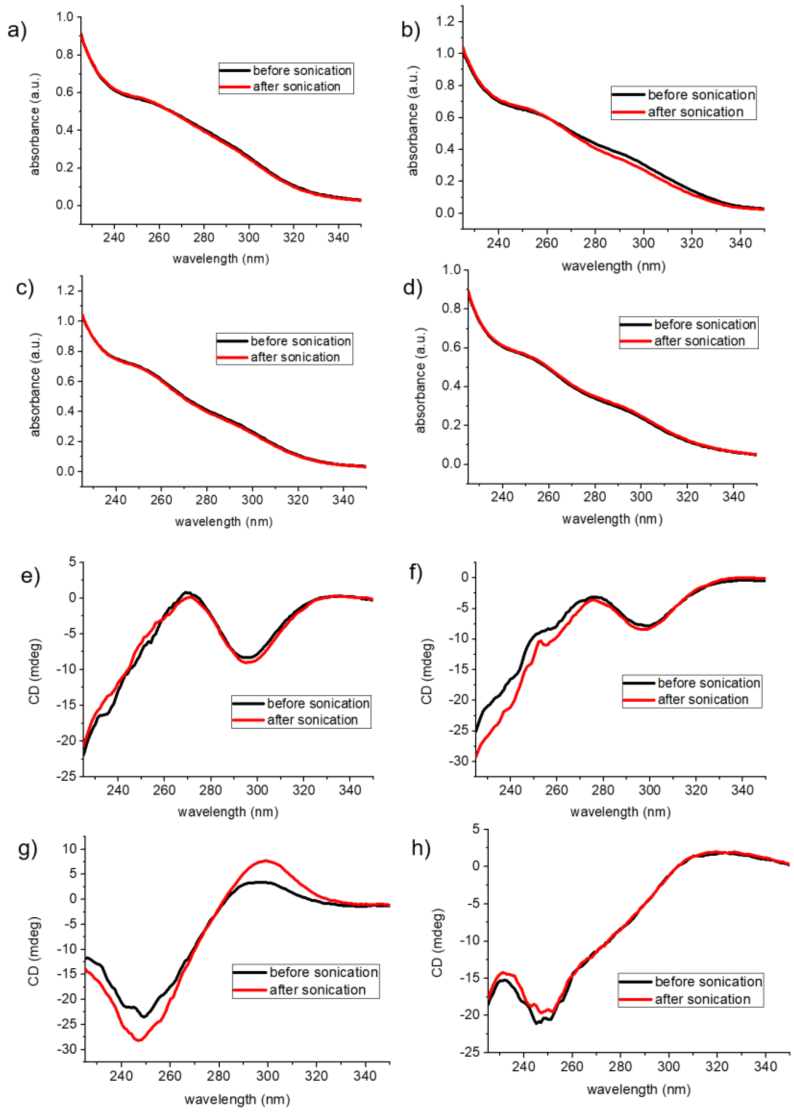


**Figure S9.** UV-Vis of (a) TTA 1, (b) TTA 2, (c) TTA 3, (d) TTA 4 at 100 µM assembled in MCH at 298 K before and after 20 s sonication. CD of (a) TTA 1, (b) TTA 2, (c) TTA 3, (d) TTA 4 at 100 µM assembled in MCH at 298 K before and after 20 s sonication.

Both UV-Vis and CD show a similar shape of spectra after sonication suggesting that the sonication doesn't change the alignment of the building blocks in the assembled state of TTA 1 - TTA 4.

## 11. Investigation of sonication influence on TTA 1 - TTA 4 by TEM

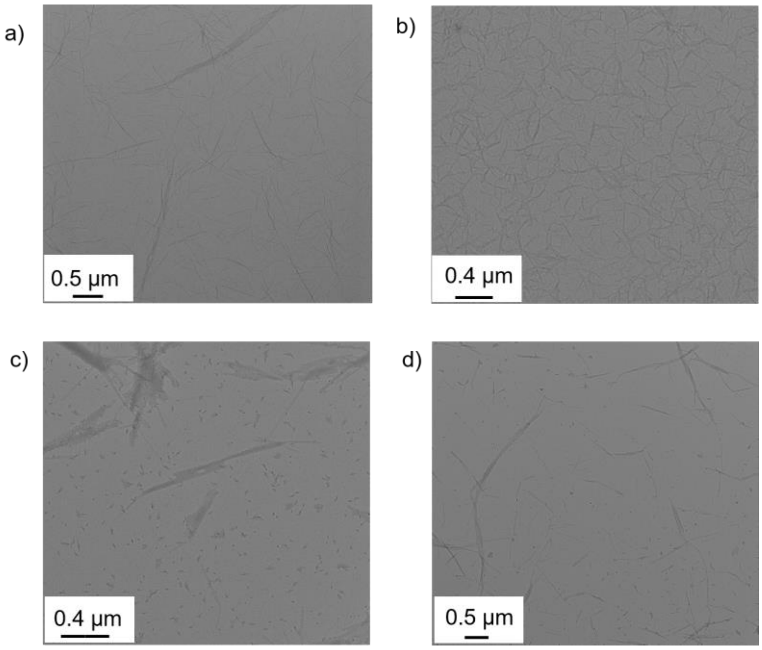


**Figure S10.** TEM of (a) TTA 1, (b) TTA 2, (c) TTA 3, (d) TTA 4 at 100 M assembled in MCH at 298 K after 20 s sonication and dried.

After sonication TTA 1 - TTA 4 become shorter fragments.

## 12. Seeded supramolecular polymerization of TTA 4 followed by CD spectroscopy.

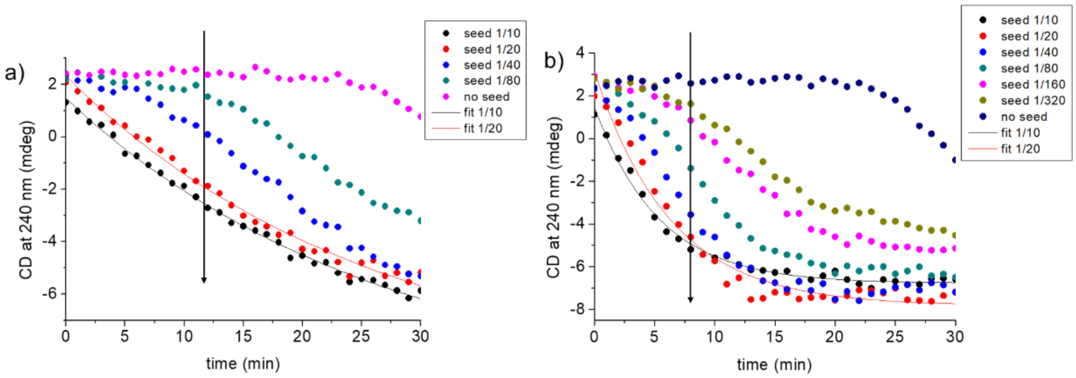


**Figure S11.** (a) CD intensity at 240 nm as a function of time after cooling TTA 4 (30 μM) in MCH from 358 K to 288 K at a rate of 10 K min$^{-1}$ prepared in the presence of various concentrations of seeds. By fitting the curve obtained for the ratio 1/10 to the integrating eq 5 in the main text, we get the activation constant $k_+ = 0.03732\ \mathrm{min}^{-1}$ with $R^2 = 0.996$. Fit the ratio 1/20 get $k_+ = 0.03853\ \mathrm{min}^{-1}$ with $R^2 = 0.990$. (b) CD intensity at 240 nm as a function of time after cooling TTA 4 (30 μM) in MCH from 358 K to 298 K at a rate of 10 K min$^{-1}$ prepared in the presence of various concentrations of seeds. By fitting the curve obtained for the ratio 1/10 to the integrating eq 5 in the main text, we get the activation constant $k_+ = 0.1869\ \mathrm{min}^{-1}$ with $R^2 = 0.990$. Fit the ratio 1/20 get $k_+ = 0.1587\ \mathrm{min}^{-1}$ with $R^2 = 0.968$.

Integrating eq 5 in the main text, we get:

$$[\mathrm{M}] = \frac{k_- k_\mathrm{d}}{k_\mathrm{a} k_+} - \left(\frac{k_- k_\mathrm{d}}{k_\mathrm{a} k_+} - [\mathrm{M}]_0\right) \exp\left(-k_+ \mathrm{t}\right) \qquad (6)$$

Where $[\mathrm{M}]_0$ is the concentration of the trapped monomeric state at the beginning. Then we can get the building block at the assembled state:

$$[\mathrm{P}] = [\mathrm{M}]_0 - [\mathrm{M}] = \left(\frac{k_- k_\mathrm{d}}{k_\mathrm{a} k_+} - [\mathrm{M}]_0\right) \exp\left(-k_+ \mathrm{t}\right) + [\mathrm{M}]_0 - \frac{k_- k_\mathrm{d}}{k_\mathrm{a} k_+} \qquad (7)$$

We assume for the CD at 240 nm, the contribution from the trapped monomeric state is $k_\mathrm{cm}[\mathrm{M}]$ and the contribution from the assembled state is $k_\mathrm{cp}[\mathrm{P}]$ where $k_\mathrm{cm}$ and $k_\mathrm{cp}$ are parameters. Then the CD value at 240 nm can be obtained as:

$$k_\mathrm{cm}[\mathrm{M}] + k_\mathrm{cp}[\mathrm{P}] = k_\mathrm{cp}[\mathrm{M}]_0 + \left(k_\mathrm{cm} - k_\mathrm{cp}\right)\frac{k_- k_\mathrm{d}}{k_\mathrm{a} k_+} + \left(k_\mathrm{cp} - k_\mathrm{cm}\right)\left(\frac{k_- k_\mathrm{d}}{k_\mathrm{a} k_+} - [\mathrm{M}]_0\right) \exp\left(-k_+ \mathrm{t}\right) \qquad (8)$$

If we let $y = k_\mathrm{cm}[\mathrm{M}] + k_\mathrm{cp}[\mathrm{P}]$, $a = k_\mathrm{cp}[\mathrm{M}]_0 + \left(k_\mathrm{cm} - k_\mathrm{cp}\right)\frac{k_- k_\mathrm{d}}{k_\mathrm{a} k_+}$, and $b = \left(k_\mathrm{cp} - k_\mathrm{cm}\right)\left(\frac{k_- k_\mathrm{d}}{k_\mathrm{a} k_+} - [\mathrm{M}]_0\right)$,

then we can simplify eq 8 to:

$$y = a + b\exp\left(-k_+ \mathrm{t}\right) \qquad (9)$$

Where $y$ is CD value, t is time, $a$ and $b$ are parameters. By fitting this integrated equation to the data above, we can get $k_+$.

**13. Seeded and hetero-seeded supramolecular polymerization of TTA 4 followed DLS**

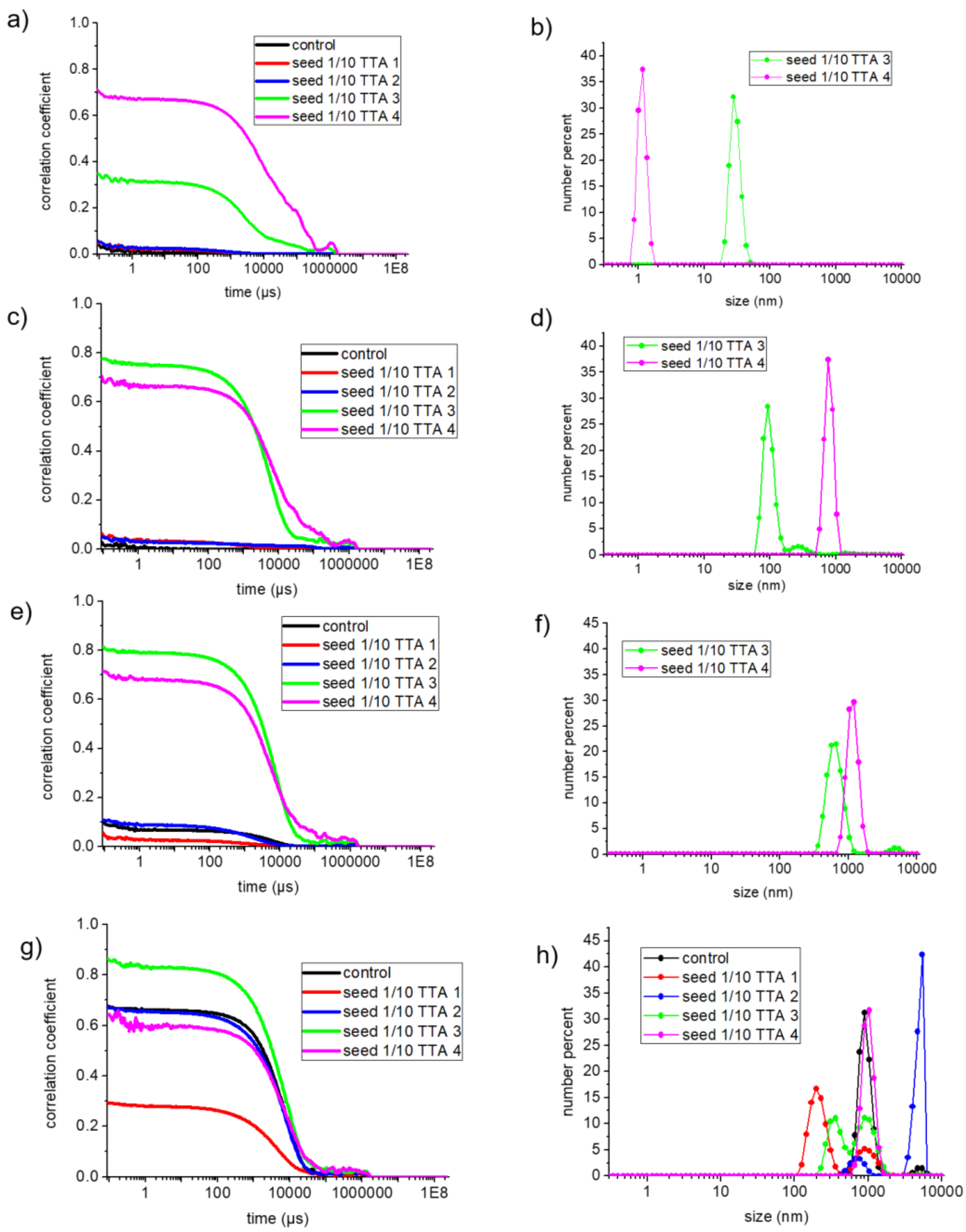


**Figure S12.** TTA 4 in MCH (30 μM) after fast cooling to 298 K was then added with TTA 1- TTA 4 seeds separately at the ratio of 1/10 while keeping the total concentration of TTA 4 at 30 μM. The control group is the TTA 4 solution without the addition of anything after cooling. Correlation coefficients were measured at (a) 1 min, (c) 8 min, (e) 16 min, and (g) 32 min after the addition of seeds. DLS results were measured at (b) 1 min, (d) 8 min, (f) 16 min, and (h) 32 min after the addition of seeds.

For the control group, the TTA 1 seeding group and the TTA 2 seeding group because of the poor correlation coefficients at first 16 min (Figure S12a, c, e), no reliable size can be measured by DLS. However, these poor correlation coefficients can indirectly support that TTA 1 and TTA 2 cannot trigger the self-assembly of TTA 4 monomer. After 32 min correlation coefficients of these three groups become good enough to get reliable DLS results (Figure S12g). At this time control group shows a peak around 1000 nm, which is consistent with assembled state. However as to TTA 1 seeding group and the TTA 2

seeding group, due to the hetero-seeding experiment followed by CD (Figure 7), there is no assembled state formed after 32 min. Therefore the peaks of these two groups are probably due to the partial clustering of TTA 1 and TTA 2 seeds.

For the TTA 3 seeding group and the TTA 4 seeding group the correlation coefficient is always good enough to get reliable DLS results (Figure S12a, c, e, g). The DLS results show that after the seeding of TTA 3 and TTA 4, the size increases gradually from 1 min to 16 min (Figure S12b, d, f), and stays at that size after 32 min (Figure S12g). These DLS results are consistent with the CD results that both TTA 4 and TTA 3 seeds can trigger the self-assembly of TTA 4 monomer. The extra peak that occurred at 32 min for TTA 3 seeding group can be probably attributed to the partial clustering of the remaining TTA 3 seeds just like the case in TTA 1 and TTA 2 seeding groups.

**14. Heating curve of TTA 1 - TTA 4 fitted to a cooperative model.**

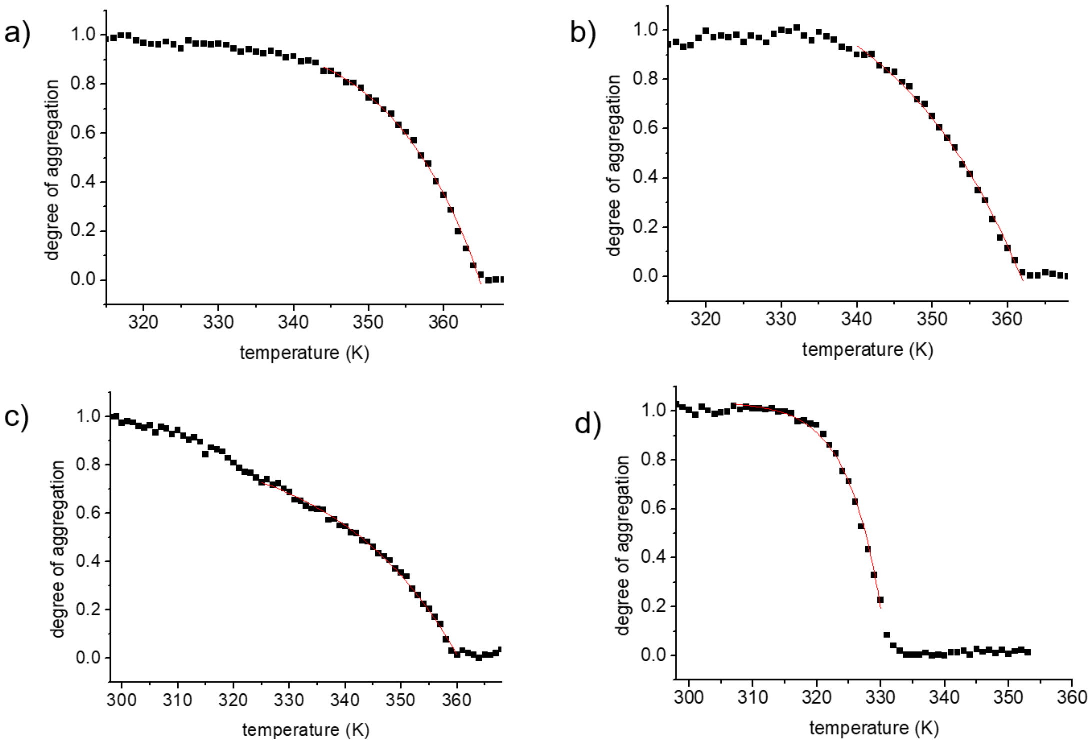


**Figure S13**. Degree of aggregation (normalized CD activity at 240 nm) as a function of the temperature during the heating of a 100 µM solution of (a) TTA 1 in MCH from 298 K to 368 K (b) TTA 2 in MCH from 298 K to 368 K (c) TTA 3 in MCH from 298 K to 368 K and (d)TTA 4 in MCH from 298 K to 358 K at a rate of 1 K min$^{-1}$. After fitting the curve to the model proposed by Meijer, Schenning et al.[3] we obtain for TTA 1 the elongation enthalpy $\Delta H_e = -95\ \mathrm{kJ\ mol^{-1}}$ and the critical elongation temperature $T_e = 366\ \mathrm{K}$, $R^2 = 0.997$, for TTA 2 the elongation enthalpy $\Delta H_e = -62\ \mathrm{kJ\ mol^{-1}}$ and the critical elongation temperature $T_e = 363\ \mathrm{K}$, $R^2 = 0.996$, for TTA 3 the elongation enthalpy $\Delta H_e = -49\ \mathrm{kJ\ mol^{-1}}$ and the critical elongation temperature $T_e = 360\ \mathrm{K}$, $R^2 = 0.996$, for TTA 4 the elongation enthalpy $\Delta H_e = -171\ \mathrm{kJ\ mol^{-1}}$ and the critical elongation temperature $T_e = 331\ \mathrm{K}$, $R^2 = 0.995$.

The significantly lower $\Delta H_e$ of TTA 4 compared to TTA 1 - TTA 3 is probably due to the larger R group. It forces the central amide groups to rotate at a larger angle to form intermolecular hydrogen bonds and this bond is stronger than the one in TTA 1 - TTA 3. This is consistent with the IR spectra of TTA 4in MCH, showing that the N-H stretch of the central amide groups is a peak centered at lower wavenumbers, while the N-H stretch of the central amide groups of TTA 1 - TTA 3 is a shoulder at relatively higher wavenumbers (Figure S2b). Although the $\Delta H_e$ of TTA 4 is significantly lower, its large R group can create a large entropy penalty after self-assembly. Thus TTA 4 fibers are easier to fragment than TTA 1 – TTA 3 fibers.

## 15. TEM investigation of morphology TTA 4 morphology triggered by TTA 3 seeds and TTA 4 seeds

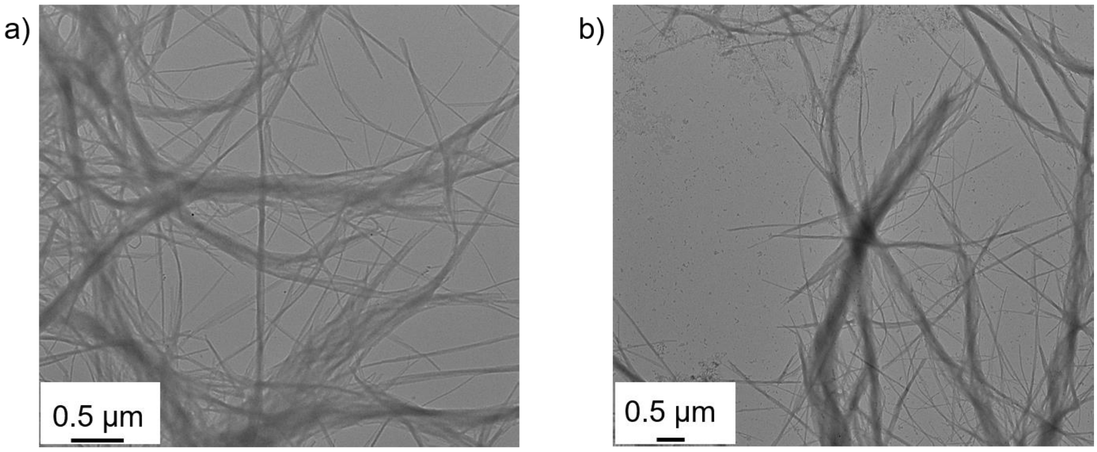


**Figure S14.** TEM of TTA 4 at 30 M in MCH at 298 K after seeding with (a) TTA 4 at the ratio 1/10 and (b) TTA 3 at the ratio 1/10 and dried.

## 16. Temperature-dependent $^1$H NMR spectroscopy of TTA 4.

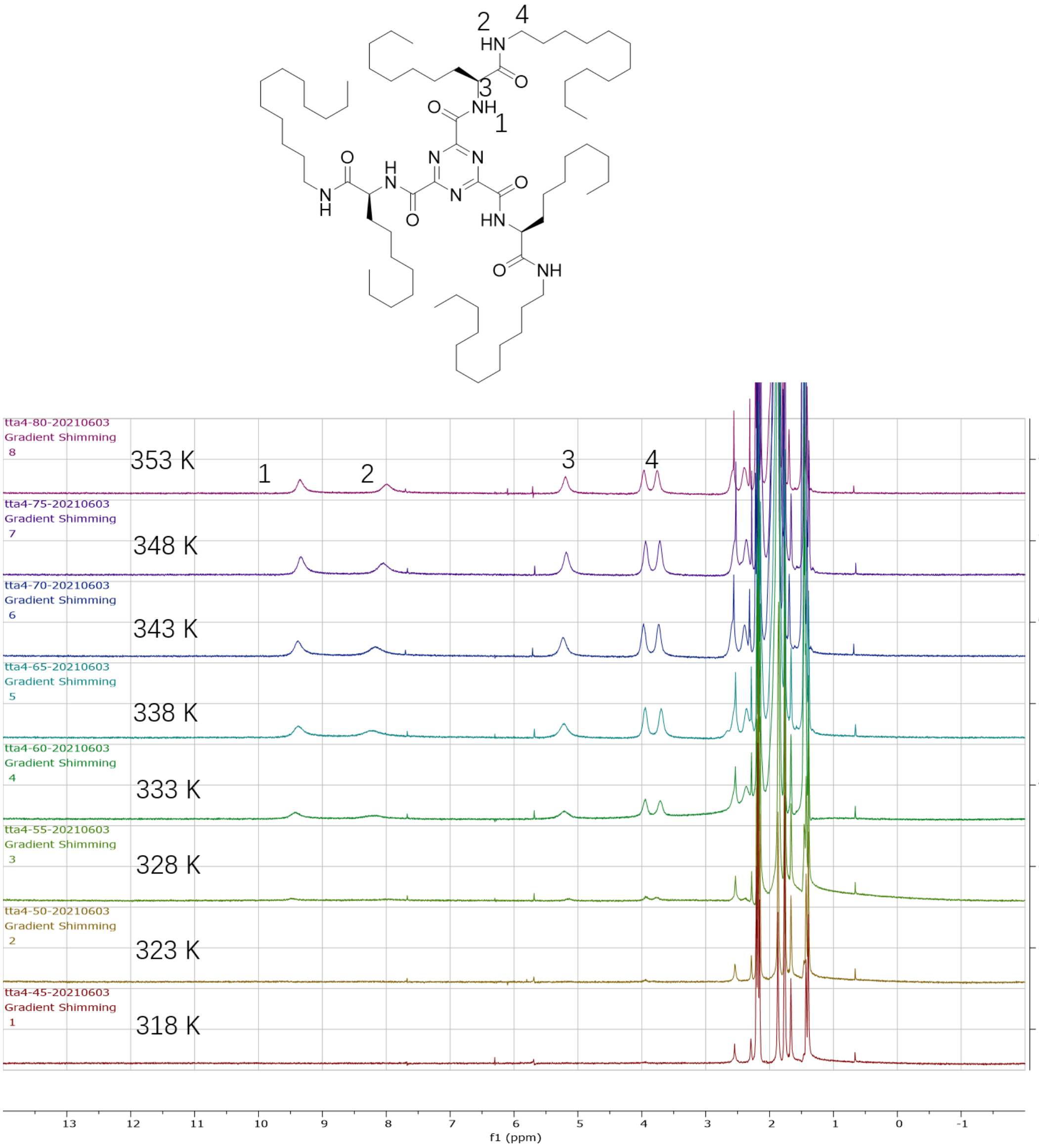


**Figure S15.** $^1$H NMR spectra of a 5.6 mM solution of TTA 4 in MCH-$d_{14}$ at various temperatures.

## 17. Synthesis

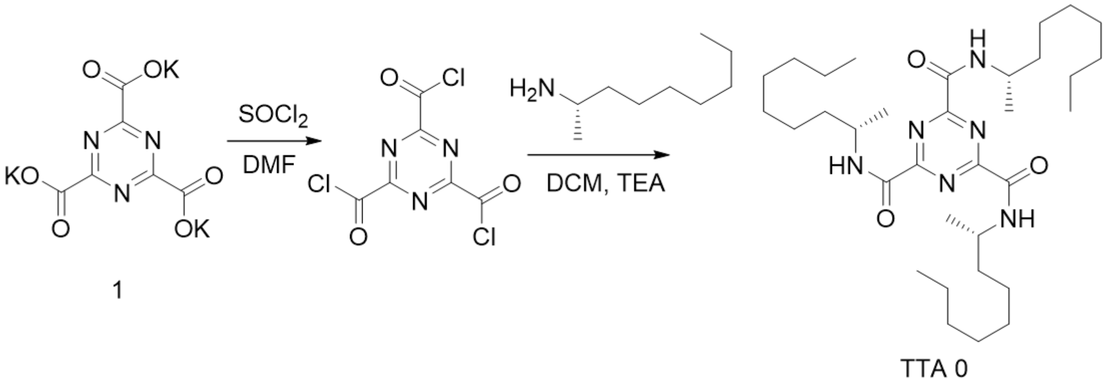


**Figure S16.** Synthetic route for the preparation of TTA 0.

***N[2],N[4],N[6]-tri((S)-nonan-2-yl)-1,3,5-triazine-2,4,6-tricarboxamide* (TTA 0)**:

Potassium 1,3,5-triazine-2,4,6-tricarboxylate (**1**) was obtained as a white powder by hydrolysis of triethyl 1,3,5-triazine-2,4,6-tricarboxylate as reported elsewhere.[5] 10 mL of $SOCl_2$ were added to 0.4 g of **1** (1.22 mmol) at r.t in a round bottom flask. Then 5 drops of anhydrous DMF were added at r.t. The solution was heated up to 50 °C and stirred for 4 h resulting in a yellowish transparent solution. The excess $SOCl_2$ was removed under reduced pressure at 40 °C (5 min). Crude 1,3,5-triazine-2,4,6-tricarbonic acid chloride was obtained as a yellow solid. The crude acid chloride was then dissolved in 10 mL of DCM and the solution was cooled with an ice bath. (R)-2-nonanamine (0.789 g, 5.49 mmol) and triethylamine (TEA) (1.113 g, 11 mmol) were dissolved in 2 mL DCM and added drop-wise (in 15 min) to the cold acid chloride solution under stirring. Then the solution was allowed to reach r.t. and was stirred for 4 h. The reaction was then quenched by addition of 1 mL of $H_2O$ and 20 mL of chloroform. The solution was washed with 0.01 M aqueous HCl and saturated aqueous $NaHCO_3$, the combined organic layers were dried with $Na_2SO_4$ and solvents were removed by rotary evaporation. The crude product was purified by silica gel column chromatography with EtOAc/$CHCl_3$ (1:2) + 1% EtOH as the eluant (the extra EtOH was found to remove the long tail during the chromatography). The product was then purified further by reverse phase chromatography on a $C_{18}$ column with MeOH/DCM (10:1) as the eluant. TTA 0 was obtained as a colorless transparent solid after removing the solvents under reduced pressure (0.314 g, 0.54 mmol, yield 44%). $^1$H NMR (400 MHz, Chloroform-*d*) δ = 8.37 (d, *J* = 8.9 Hz, 3H), 4.23 (hept, *J* = 6.9 Hz, 3H), 1.61 (dhept, *J* = 20.9, 6.9 Hz, 6H), 1.40 – 1.18 (m, 39H), 0.86 (t, *J* = 6.6 Hz, 9H). $^{13}$C NMR (101 MHz, $CDCl_3$) δ = 166.90, 159.29, 47.09, 36.63, 31.94, 29.53, 29.33, 26.41, 22.78, 20.72, 14.22. MS (ESI+) (m/z) calculated for $[M + Na]^+$: 611.4619, found: 611.6166.

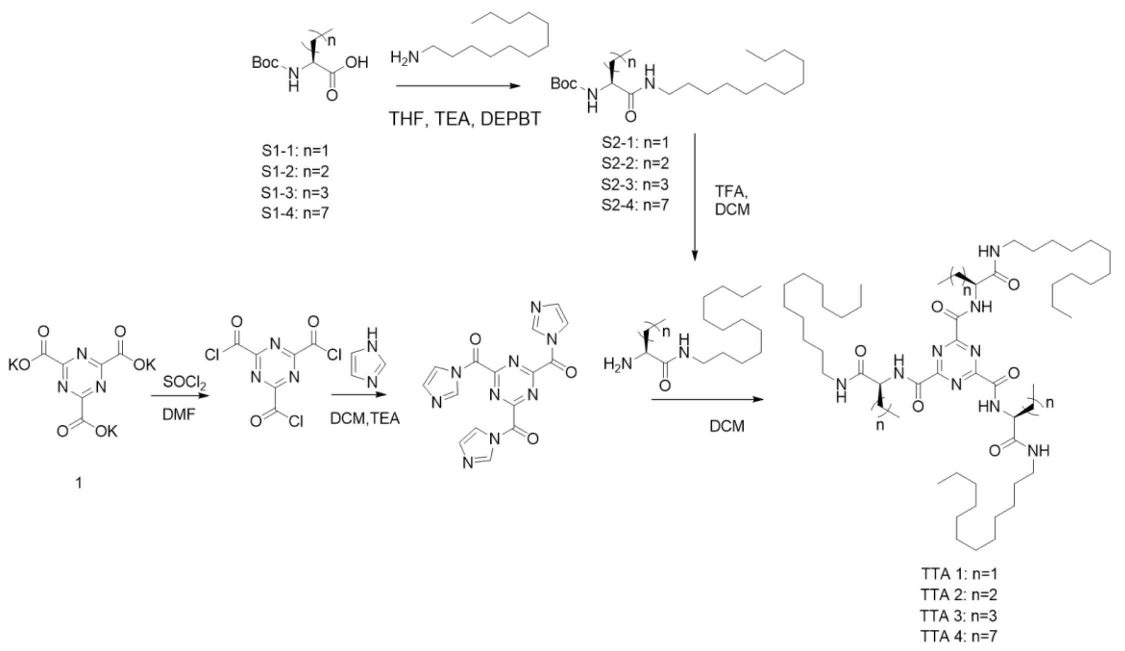

**Figure S17.** Synthetic route for the preparation of TTA 1-4.

***tert-butyl (S)-(1-(dodecylamino)-1-oxobutan-2-yl)carbamate* (S2-1):**

Boc-L-Abu-OH (**S1-1**, 1.089 g, 5.36 mmol) and 3-(Diethoxyphosphoryloxy)-1,2,3-benzotriazin-4(3H)-one (DEPBT) (1.763 g, 5.89 mmol) were dissolved in 18 mL of THF in a round bottom flask at r.t under stirring. TEA (1.035 g, 10.71 mmol) was added and the solution was stirred at r.t. for 40 min to activate the carboxylic acid. Then dodecylamine (1.092 g, 5.89 mmol) was added and the solution was stirred at r.t. for 12 h., the mixture was purified by column silica gel column chromatography with EtOAc/heptane (1:4) as the eluant. **S2-1** was obtained as a white solid after removing the solvents under reduced pressure (1.920 g, 5.19 mmol, yield 97%). $^{1}$H NMR (400 MHz, $CDCl_3$) δ = 6.25 (s, 1H), 5.12 (s, 1H), 3.97 (d, *J* = 8.1 Hz, 1H), 3.43 - 3.04 (m, 2H), 1.91 - 1.55 (m, 2H), 1.42 (m, 11H), 1.25 (m, 18H), 0.89 (m, 6H). $^{13}$C NMR (101 MHz, $CDCl_3$) δ = 172.00, 155.90, 80.00, 55.98, 39.62, 32.04, 29.77, 29.75, 29.71, 29.70, 29.66, 29.47, 29.41, 28.45, 27.00, 25.96, 22.81, 14.24, 10.15. MS (ESI+) (m/z) calculated for $[M + Na]^+$: 393.3088, found: 393.2949.

***tert-butyl (S)-(1-(dodecylamino)-1-oxopentan-2-yl)carbamate* (S2-2):**

**S2-2** was synthesized according to the same protocol used for the synthesis of **S2-1**. Except that Boc-L-Nva-OH (**S1-2**, 1.164g, 5.36 mmol) was used instead of **S1-1**. Purification was carried out by silica gel column chromatography with EtOAc/heptane (1:6) as the eluant. **S2-2** was obtained as a white solid after removing the solvents under reduced pressure (1.963 g, 5.10 mmol, yield 95%). $^{1}$H NMR (400 MHz, $CDCl_3$) δ = 6.13 (d, *J* = 7.1 Hz, 1H), 5.01 (s, 1H), 4.00 (d, *J* = 7.5 Hz, 1H), 3.40 - 3.13 (m, 2H), 1.89 - 1.54 (m, 2H), 1.43 (m, 11H), 1.25 (m, 20H), 0.89 (m, 6H). $^{13}$C NMR (101 MHz, $CDCl_3$) δ = 172.22, 155.89, 80.07, 54.66, 39.64, 34.80, 32.05, 29.78, 29.76, 29.72, 29.67, 29.48, 29.43, 29.42, 28.46, 27.00, 22.82, 19.07, 14.25, 13.92. MS (ESI+) (m/z) calculated for $[M + Na]^+$: 407.3244; found: 407.2264.

***tert-butyl (S)-(1-(dodecylamino)-1-oxohexan-2-yl)carbamate* (S2-3):**

**S2-3** was synthesized according to the same protocol used for the synthesis of **S2-1**. Except that Boc-L-Nle-OH (**S1-3**, 1.239 g, 5.36 mmol) was used instead of **S1-1**. Purification was carried out by silica gel column chromatography with EtOAc/heptane (1:6) as the eluant. **S2-3** was obtained as a white solid after removing the solvents under reduced pressure (0.937 g, 2.35 mmol, yield 44%). $^{1}$H NMR (400 MHz,

$CDCl_3$) δ = 6.08 (t, *J* = 5.7 Hz, 1H), 5.00 (s, 1H), 3.99 (q, *J* = 7.3 Hz, 1H), 3.23 (qd, *J* = 6.9, 3.4 Hz, 2H), 1.91 - 1.53 (m, 2H), 1.44 (m, 11H), 1.26 (m, *J* = 13.1 Hz, 22H), 0.95 - 0.80 (m, 6H). $^{13}C$ NMR (101 MHz, $CDCl_3$) δ 172.19, 155.87, 80.10, 54.86, 39.64, 32.41, 32.06, 29.79, 29.76, 29.73, 29.68, 29.49, 29.43, 28.47, 27.92, 27.01, 22.83, 22.58, 14.26, 14.06. MS (ESI+) (m/z) calculated for [M + Na]$^+$: 421.3401; found: 421.2928.

***tert-butyl (S)-(1-(dodecylamino)-1-oxodecan-2-yl)carbamate* (S2-4):**

**S2-4** was synthesized according to the same protocol used for the synthesis of **S2-1**. Except that Boc-L-Adc-OH (**S1-4**, 0.548 g, 1.91 mmol) was used instead of **S1-1,** and the reaction was performed on a smaller scale (DEPBT (0.628 g, 2.10 mmol), THF 6.4 mL, TEA (0.369 g, 3.65 mmol), dodecylamine (0.389 g, 2.10 mmol)). Purification was carried out by silica gel column chromatography EtOAc/heptane (1:8). 0.780 g of **S2-4** was obtained as a white solid after removing the solvents under reduced pressure (, 1.72 mmol, yield 90%). $^1H$ NMR (400 MHz, $CDCl_3$) δ = 6.15 (t, *J* = 5.7 Hz, 1H), 5.18 – 4.92 (m, 1H), 3.99 (q, *J* = 7.5 Hz, 1H), 3.38 – 3.07 (m, 2H), 1.87 – 1.52 (m, 2H), 1.43 (m, 11H), 1.25 (m, 30H), 0.87 (m, 6H). $^{13}C$ NMR (101 MHz, $CDCl_3$) δ = 172.21, 155.86, 80.03, 54.86, 39.62, 32.77, 32.05, 31.97, 29.79, 29.76, 29.74, 29.69, 29.57, 29.51, 29.49, 29.44, 29.34, 28.65, 28.46, 27.01, 25.78, 22.82, 22.78, 14.25, 14.22. MS (ESI+) (m/z) calculated for [M + Na]$^+$: 477.4027, found: 477.3875.

***$N^2$,$N^4$,$N^6$-tris((S)-1-(dodecylamino)-1-oxobutan-2-yl)-1,3,5-triazine-2,4,6-tricarboxamide* (TTA 1):**

0.964 g of **S2-1** (2.60 mmol) was dissolved in 26 mL of DCM in a round bottom flask. 8.7 mL of trifluoroacetic acid (TFA,) was added drop-wise (15 min) to the solution. The solution was stirred at r.t. for 1h and quenched with 10 mL of $H_2O$. The solution was then washed with $NaHCO_3$ and the combined organic layers were dried with $Na_2SO_4$. The crude amine was obtained by removing the solvent under reduced pressure. In parallel, 2 mL of $SOCl_2$ was added to **1** (0.246 g, 0.75 mmol) in a round bottom flask. Then 2 drops of DMF were added. The solution was stirred at 50 °C for 4 h. The excess $SOCl_2$ was then removed under reduced pressure at 40 °C (5min) and the crude 1,3,5-Triazine-2,4,6-tricarbonic acid chloride was obtained as a yellow solid. The crude acid chloride was then dissolved in 5 mL of DCM and cooled to 0°C with an ice bath. 0.307 g of imidazole (4.51 mmol) and 0.684 g of TEA (6.76 mmol) were dissolved in 3 mL of DCM and added drop-wise (15 min) to the crude acid chloride solution. Imidazole reacts with the remaining trace amount of $SOCl_2$, which renders the mixture easier to purify. The acid chloride can form active amide with imidazole which can be substituted by the crude amine. The solution was stirred at r.t. for 4 h and a yellow precipitate is obtained. The crude amine dissolved in 4 mL of DCM was then added drop-wise (15 min) at r.t. The solution was stirred at r.t. for further 12 h. The solution was diluted with 10 mL $CHCl_3$ and washed with $H_2O$. After drying the combined organic layers with $Na_2SO_4$, purification was carried out by silica gel column chromatography with EtOAc/$CHCl_3$ (2:1) as the first eluant. Then the eluant was changed to MeOH/DCM (1:40) to collect the target compound. The product was dried under reduced pressure and the resulting solid was gently washed 3 times with 1 mL of DCM. TTA 1 was obtained as a white solid after drying under reduced pressure (0.235 g, 0.24 mmol, yield 32%). $^1H$ NMR (400 MHz, $CDCl_3$) δ = 9.01 - 8.65 (m, 3H), 6.85 (s, 3H), 4.50 (q, *J* = 7.5 Hz, 3H), 3.21 (m, 6.7 Hz, 6H), 1.92 (m, 6H), 1.49 (q, *J* = 7.1 Hz, 6H), 1.25 (m, 54H), 0.92 (m, 18H). $^{13}C$ NMR (101 MHz, $CDCl_3$) δ = 170.61, 166.85, 160.22, 55.87, 40.02, 32.05, 29.81, 29.79, 29.77, 29.73, 29.68, 29.60, 29.49, 29.47, 27.13, 25.64, 22.82, 14.24, 10.43. MS (ESI+) (m/z) calculated for [M + Na]$^+$: 992.7611, found: 992.6796.

***$N^2$,$N^4$,$N^6$-tris((S)-1-(dodecylamino)-1-oxopentan-2-yl)-1,3,5-triazine-2,4,6-tricarboxamide* (TTA 2):**

**TTA 2** was synthesized according to the protocol of **TTA 1**. **S1-2** amount was 1.037g, 2.7 mmol. All the other compound were used as the same amount as the protocol of **TTA 1**. Purification was carried out

by silica gel column chromatography EtOAc/$CHCl_3$ (1:1) when the second fraction begins to come out, change to EtOAc/$CHCl_3$ (2:1) to collect all the second fraction. The product was dried under reduced pressure. Then gently washed it with DCM 1 mL 3 times. **TTA 2** was obtained as a white solid after drying under reduced pressure (0.337 g, 0.33 mmol, yield 44%). $^1$H NMR (400 MHz, $CDCl_3$) δ = 9.19 - 8.55 (m, 3H), 6.85 (m, 3H), 4.58 (q, *J* = 7.7 Hz, 3H), 3.23 (m, 6H), 2.10 - 1.75 (m, 6H), 1.51 (p, *J* = 7.1 Hz, 6H), 1.39 (tt, *J* = 9.2, 4.6 Hz, 6H), 1.25 (m, 54H), 0.90 (m, 18H). $^{13}$C NMR (101 MHz, $CDCl_3$) δ = 170.83, 166.84, 159.99, 54.47, 40.10, 34.45, 32.15, 32.06, 29.90, 29.88, 29.81, 29.79, 29.77, 29.74, 29.57, 29.50, 29.47, 27.13, 22.92, 22.83, 19.34, 19.26, 14.34, 14.25, 13.95, 13.87. MS (ESI+) (m/z) calculated for $[M + Na]^+$: 1034.8080, found: 1034.5807.

***$N^2$,$N^4$,$N^6$-tris((S)-1-(dodecylamino)-1-oxohexan-2-yl)-1,3,5-triazine-2,4,6-tricarboxamide*** **(TTA 3):**

**TTA 3** was synthesized according to the protocol of **TTA 1**. **S1-3** amount was 0.918 g, 2.30 mmol. To deprotect **S1-3**, 23 mL DCM and 7.7 mL TFA were used. To get the acid chloride, compound **1** (0.210 g, 0.64 mmol), $SOCl_2$ (1.7 mL) and 2 drops of DMF were used. After removing $SOCl_2$ crude acid chloride was dispersed in 4.3 mL DCM. Imidazole (0.261 g, 3.83 mmol) and TEA (0.583 g, 5.76 mmol) were dissolved in 2.6 mL DCM. The crude amine was dissolved in 3.4 mL DCM. Purification was carried out by silica gel column chromatography EtOAc/$CHCl_3$ (1:1). The product was dried under reduced pressure. Then gently washed it with DCM 1 mL 3 times. TTA 3 was obtained as a white solid after drying under reduced pressure (0.108 g, 0.10 mmol, yield 16%). $^1$H NMR (400 MHz, $CDCl_3$) δ = 9.03 - 8.49 (m, 3H), 6.76 (s, 3H), 4.56 (q, *J* = 7.6 Hz, 3H), 3.23 (m, 6H), 1.90 (m, 6H), 1.51 (p, *J* = 7.0 Hz, 6H), 1.42 - 1.15 (m, 66H), 0.87 (m, 18H). $^{13}$C NMR (101 MHz, $CDCl_3$) δ = 170.80, 166.84, 159.97, 54.63, 40.08, 32.14, 32.05, 29.81, 29.78, 29.77, 29.75, 29.68, 29.57, 29.49, 29.48, 28.08, 27.15, 22.82, 22.53, 14.24, 14.03. MS (ESI+) (m/z) calculated for $[M + Na]^+$: 1076.8550, found: 1076.8058.

***$N^2$,$N^4$,$N^6$-tris((S)-1-(dodecylamino)-1-oxodecan-2-yl)-1,3,5-triazine-2,4,6-tricarboxamide*** **(TTA 4):**

**TTA 4** was synthesized according to the protocol of **TTA 1**. **S1-4** amount was 0.709 g, 1.56 mmol. To deprotect **S1-4**, 14 mL DCM and 4.8 mL TFA were used. To get the acid chloride, compound **1** (0.142 g, 0.43 mmol), $SOCl_2$ (1.2 mL) and 2 drops of DMF were used. After removing $SOCl_2$ crude acid chloride was dispersed in 2.9 mL DCM. Imidazole (0.177 g, 2.60 mmol) and TEA (0.395 g, 3.90 mmol) were dissolved in 1.7 mL DCM. The crude amine was dissolved in 2.3 mL DCM. Purification was carried out by silica gel column chromatography EtOAc/DCM (1:2). The product was dried under reduced pressure. Then gently washed it with DCM 1 mL 3 times. **TTA 4** was obtained as a white solid after drying under reduced pressure (0.196 g, 0.16 mmol, yield 37%). $^1$H NMR (400 MHz, $CDCl_3$) δ = 8.95 - 8.57 (m, 3H), 6.62 (s, 3H), 4.56 (q, *J* = 7.5 Hz, 3H), 3.25 (m, 6H), 1.90 (m, 6H), 1.51 (p, *J* = 7.1 Hz, 6H), 1.26 (m, 90H), 0.92 - 0.77 (m, 18H). $^{13}$C NMR (101 MHz, $CDCl_3$) δ = 170.75, 166.82, 159.87, 54.70, 40.10, 32.44, 32.06, 31.99, 29.83, 29.80, 29.77, 29.60, 29.51, 29.50, 29.42, 27.16, 26.04, 22.83, 22.79, 14.25, 14.22. MS (ESI+) (m/z) calculated for $[M + Na]^+$: 1245.0428, found: 1245.0038.

## 18. Characterization

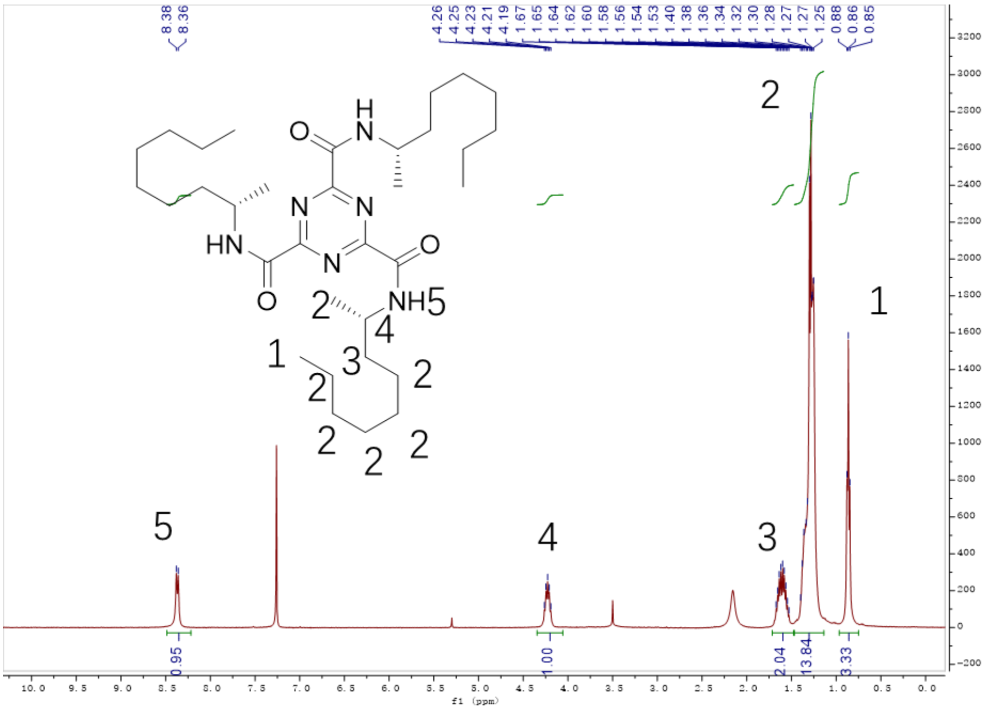


**Figure S18.** $^{1}H$ NMR of TTA 0 (400 MHz, 298 K, $CDCl_3$).

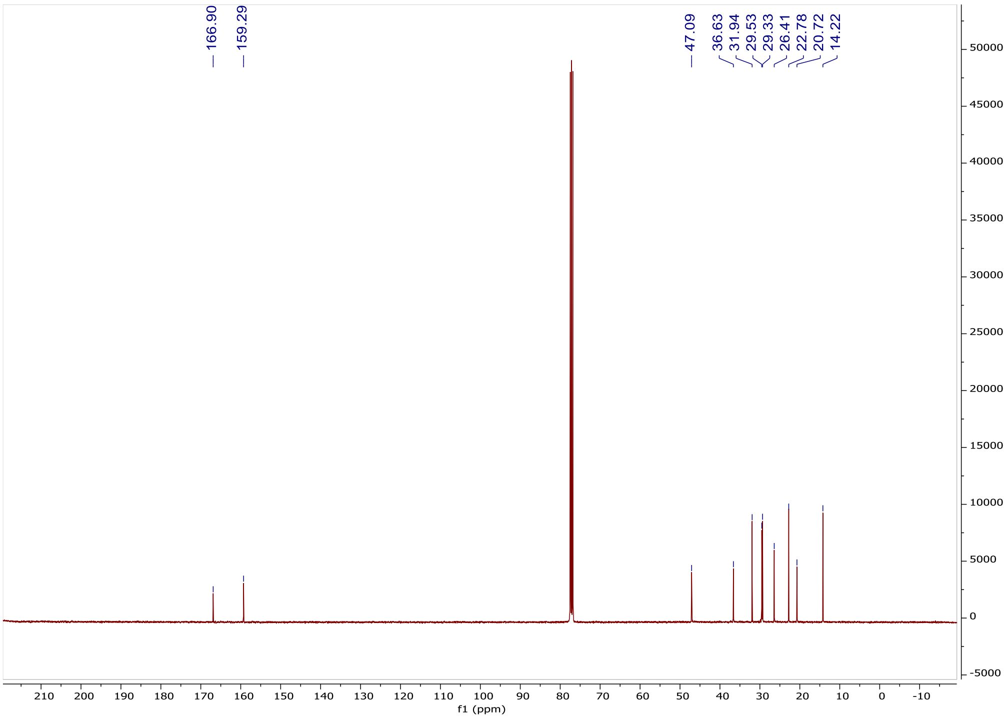


**Figure S19.** $^{13}C$ NMR of TTA 0 (101 MHz, 298 K, $CDCl_3$).

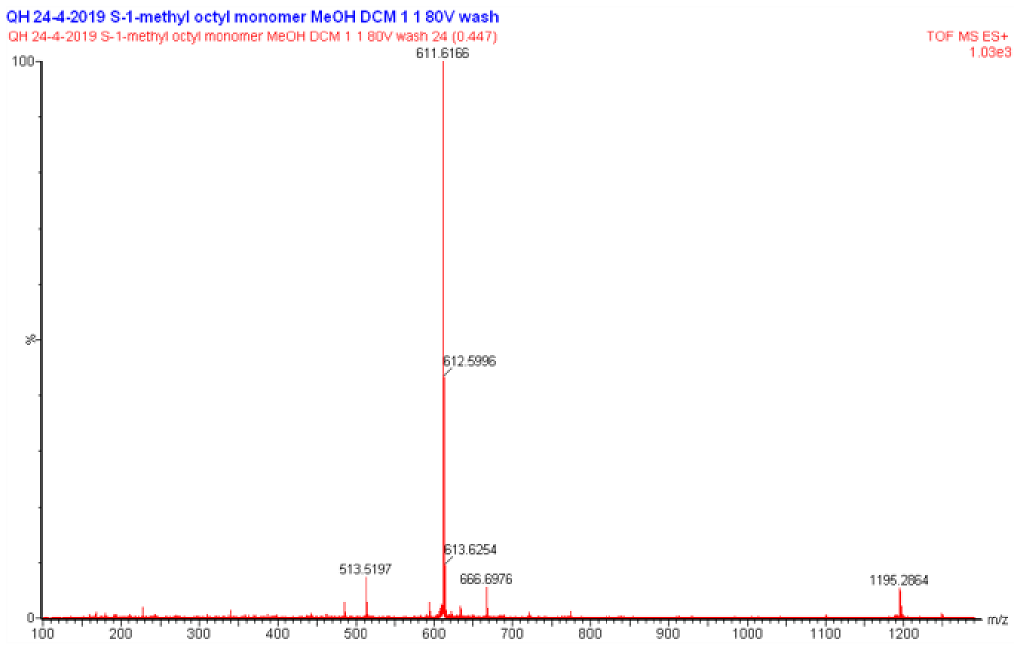


**Figure S20.** ESI-mass spectrum of TTA 0 ([M + Na]+ 611.6166).

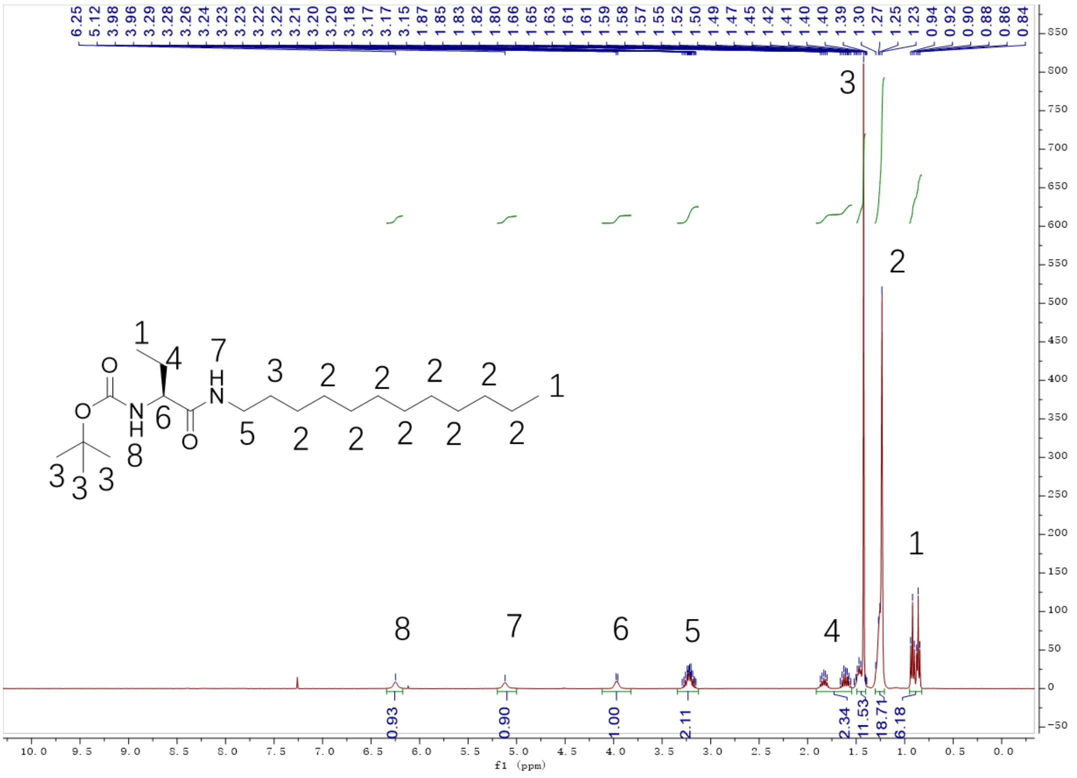

**Figure S21.** $^1$H NMR of **S2-1** (400 MHz, 298 K, $CDCl_3$).

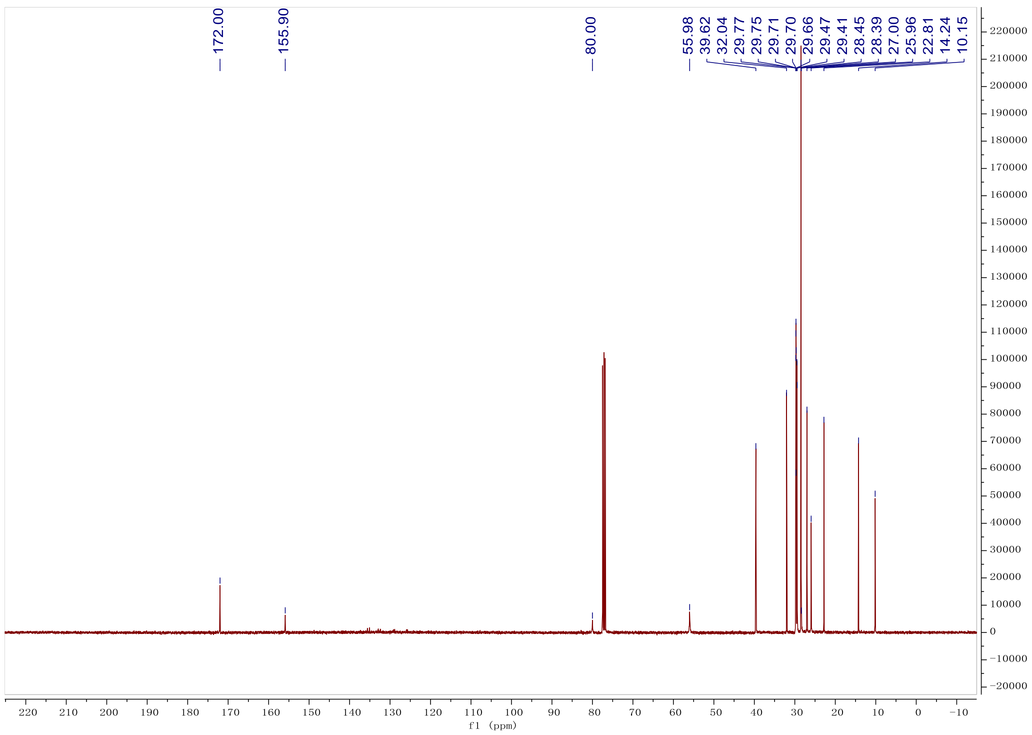


**Figure S22.** $^{13}C$ NMR of S**2-1** (101 MHz, 298 K, $CDCl_3$).

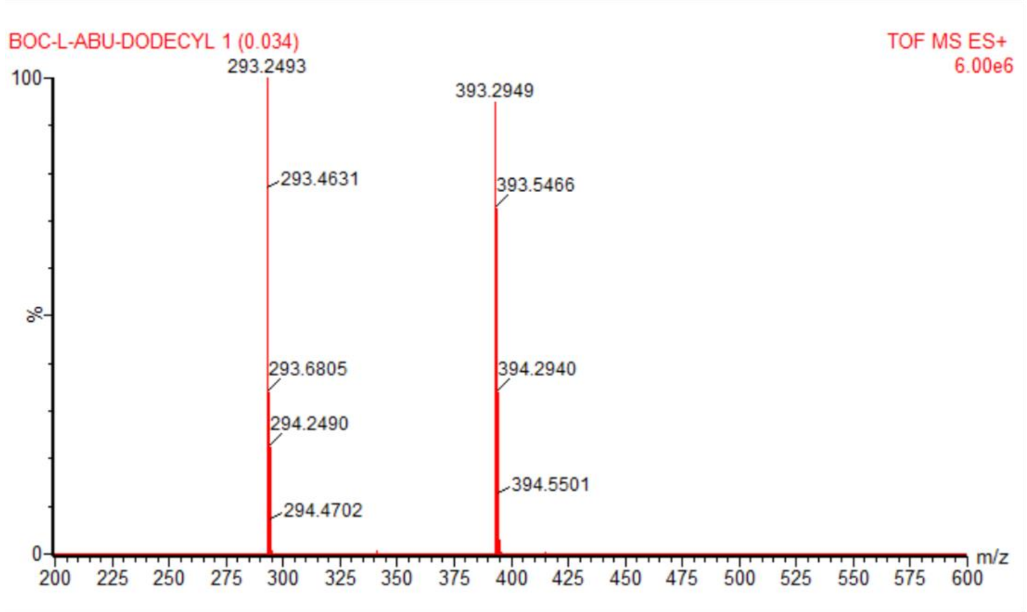


**Figure S23.** ESI-mass spectrum of **S2-1** ([M + Na]+ 393.2949).

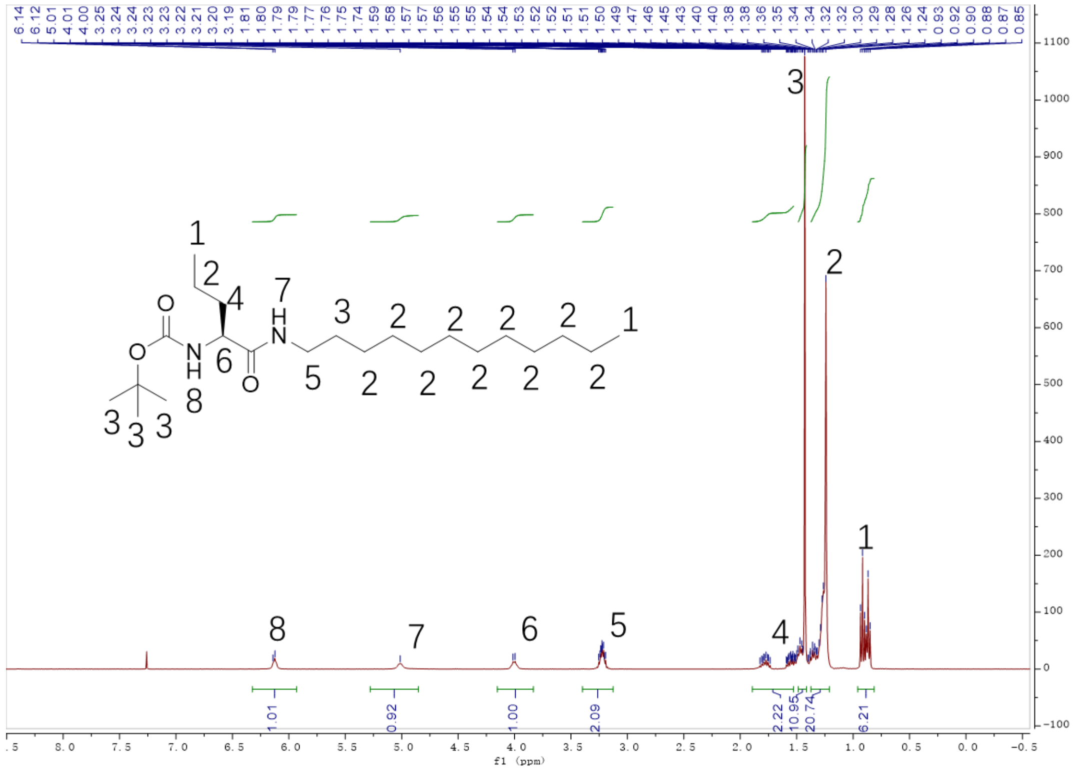


**Figure S24.** $^{1}H$ NMR of **S2-2** (400 MHz, 298 K, $CDCl_3$).

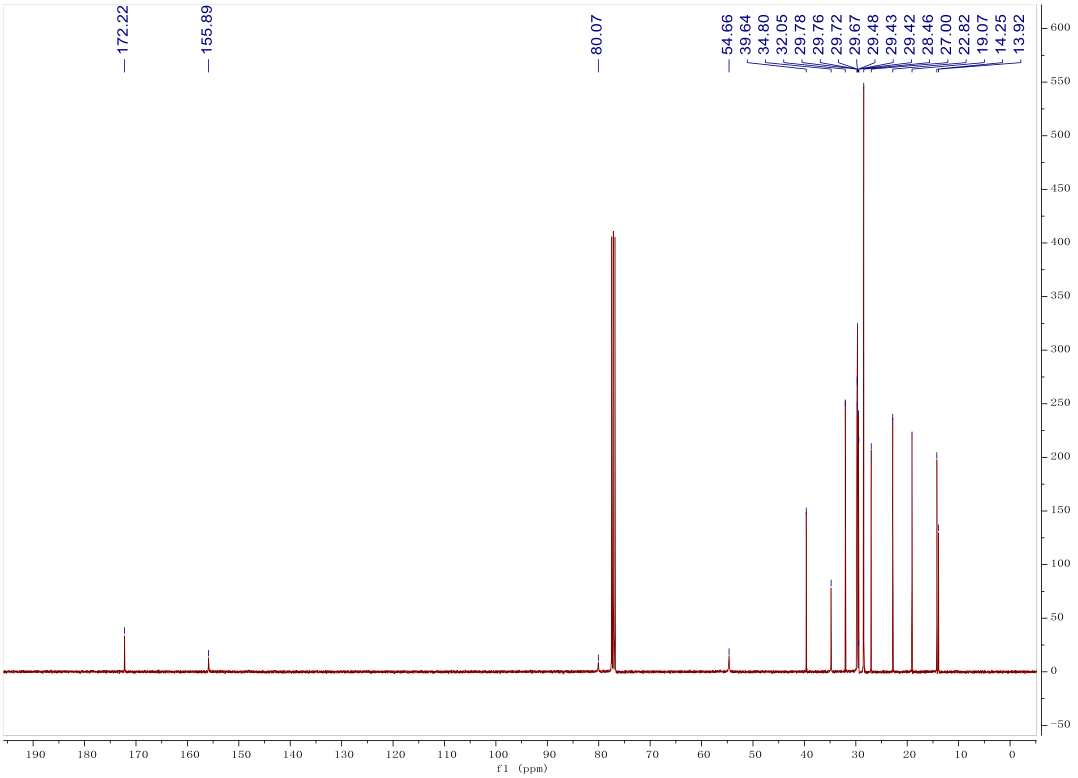


**Figure S25.** $^{13}C$ NMR of **S2-2** (101 MHz, 298 K, $CDCl_3$).

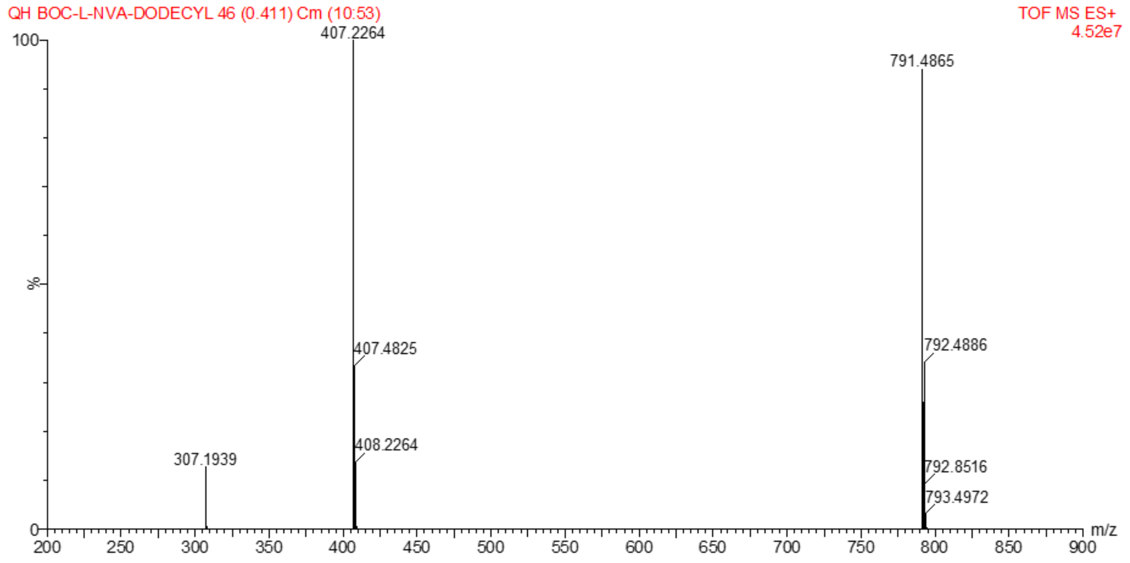


**Figure S26.** ESI-mass spectrum of **S2-2** ([M + Na]+ 407.2264).

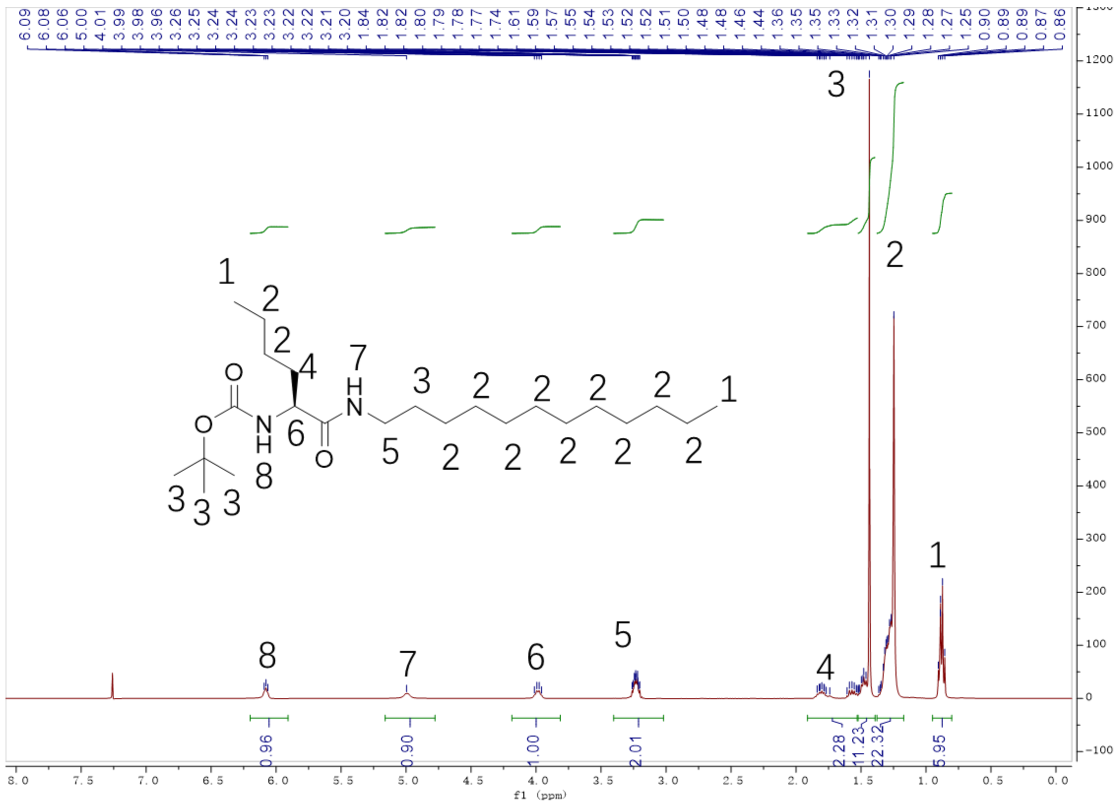


**Figure S27.** $^{1}$H NMR of **S2-3** (400 MHz, 298 K, $CDCl_3$).

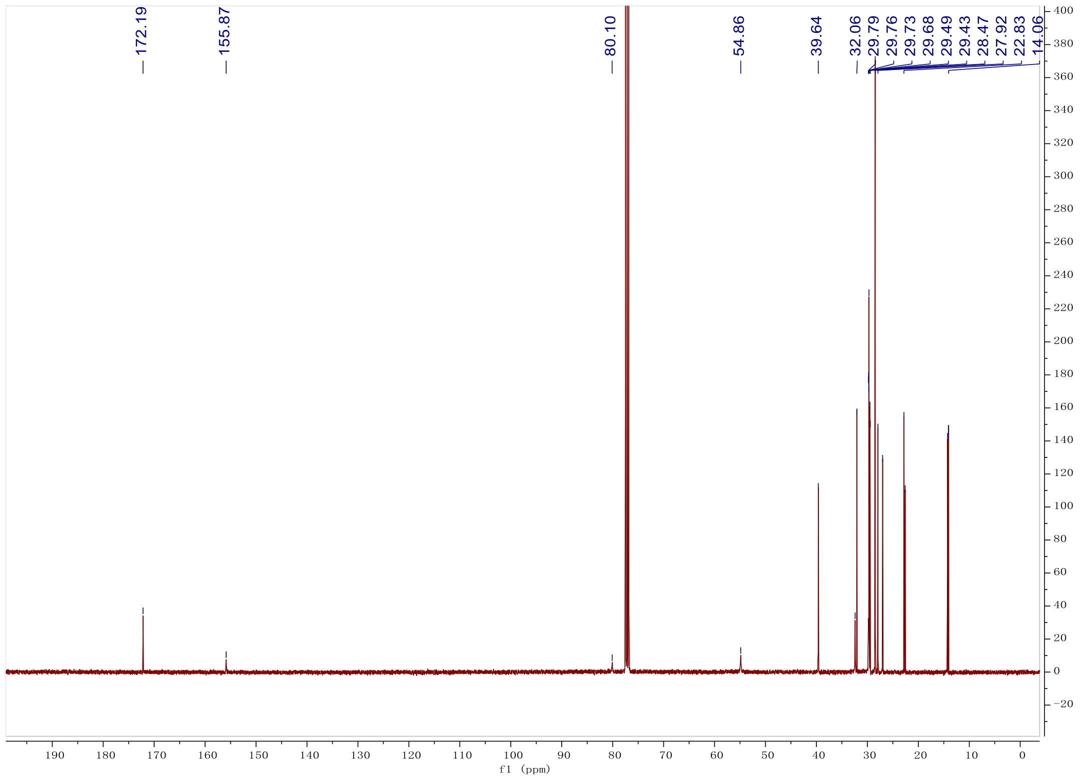


**Figure S28.** $^{13}C$ NMR of **S2-3** (101 MHz, 298 K, $CDCl_3$).

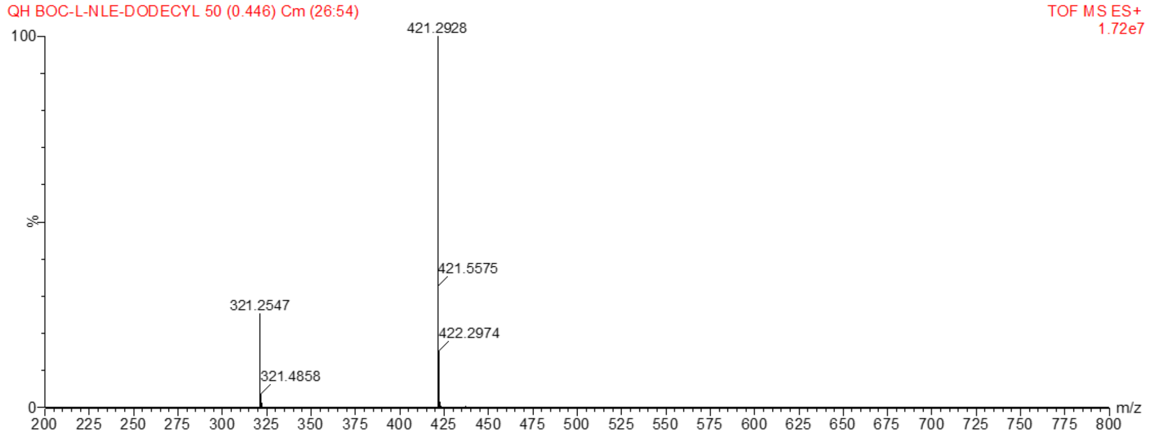


**Figure S29.** ESI-mass spectrum of **S2-3** ([M + Na]$^+$ 421.2928).

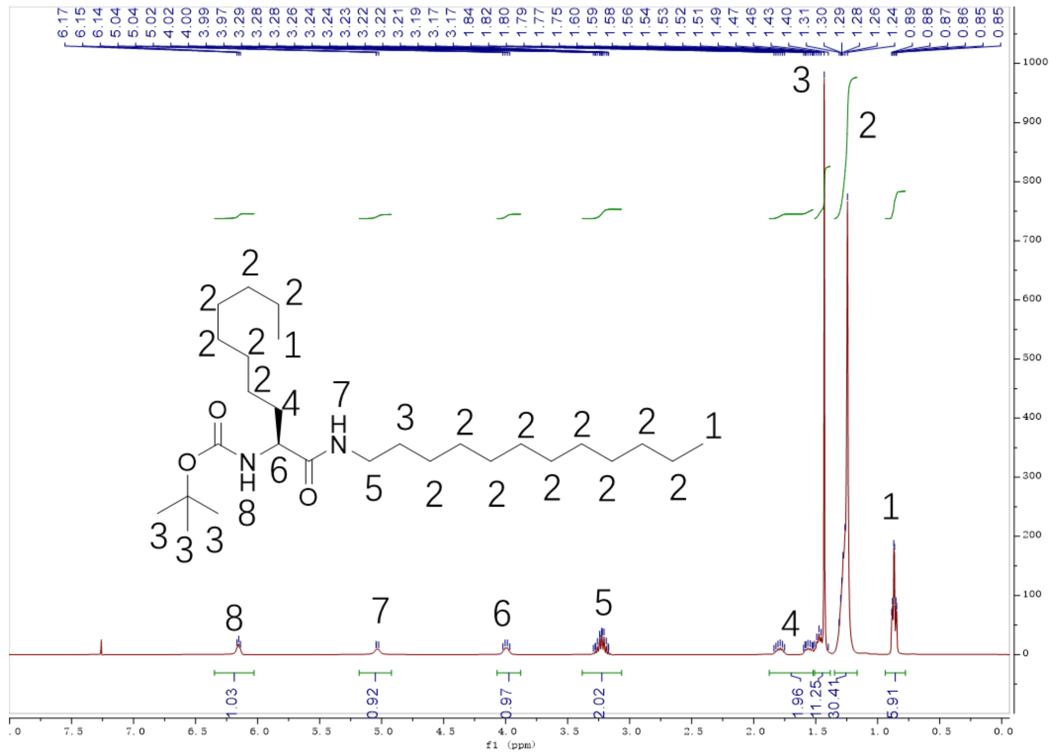

**Figure S30.** $^{1}H$ NMR of **S2-4** (400 MHz, 298 K, $CDCl_3$).

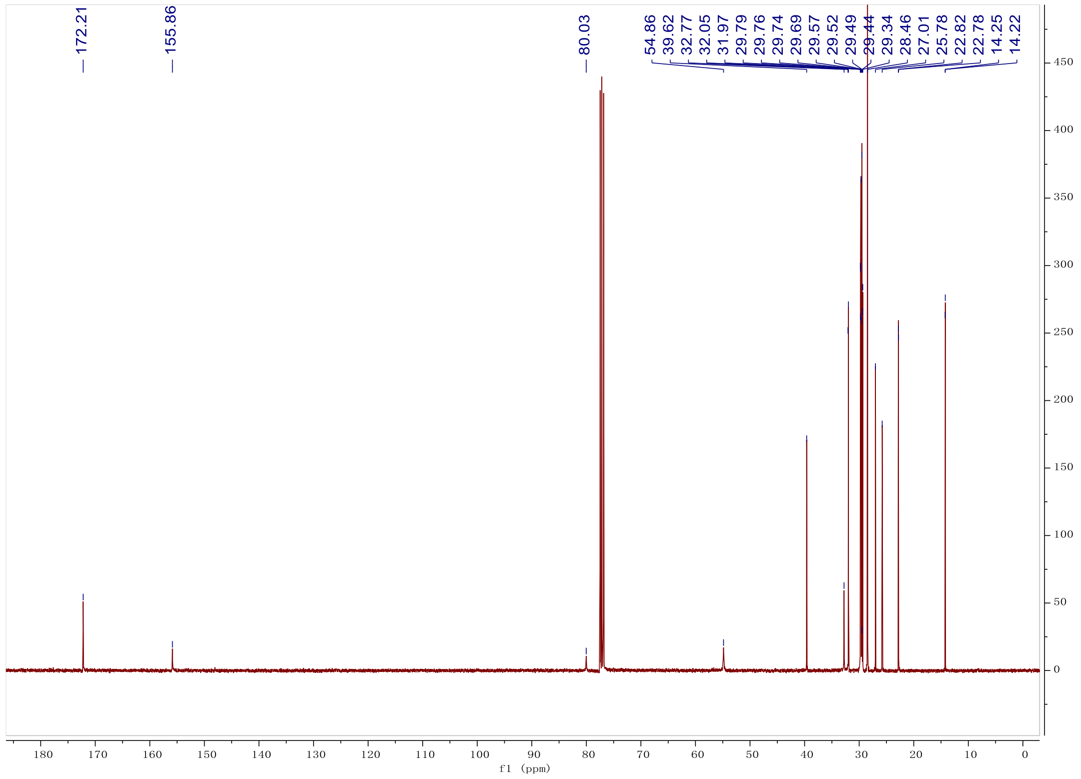

**Figure S31.** $^{13}C$ NMR of **S2-4** (101 MHz, 298 K, $CDCl_3$).

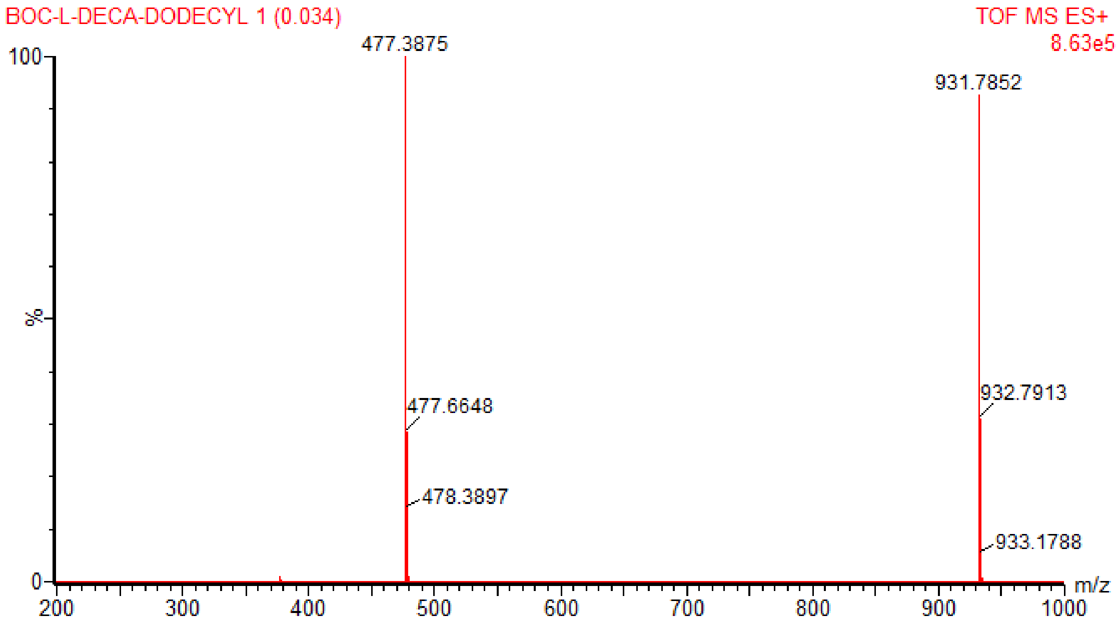


**Figure S32.** ESI-mass spectrum of **S2-4** ([M + Na]+ 477.3875).

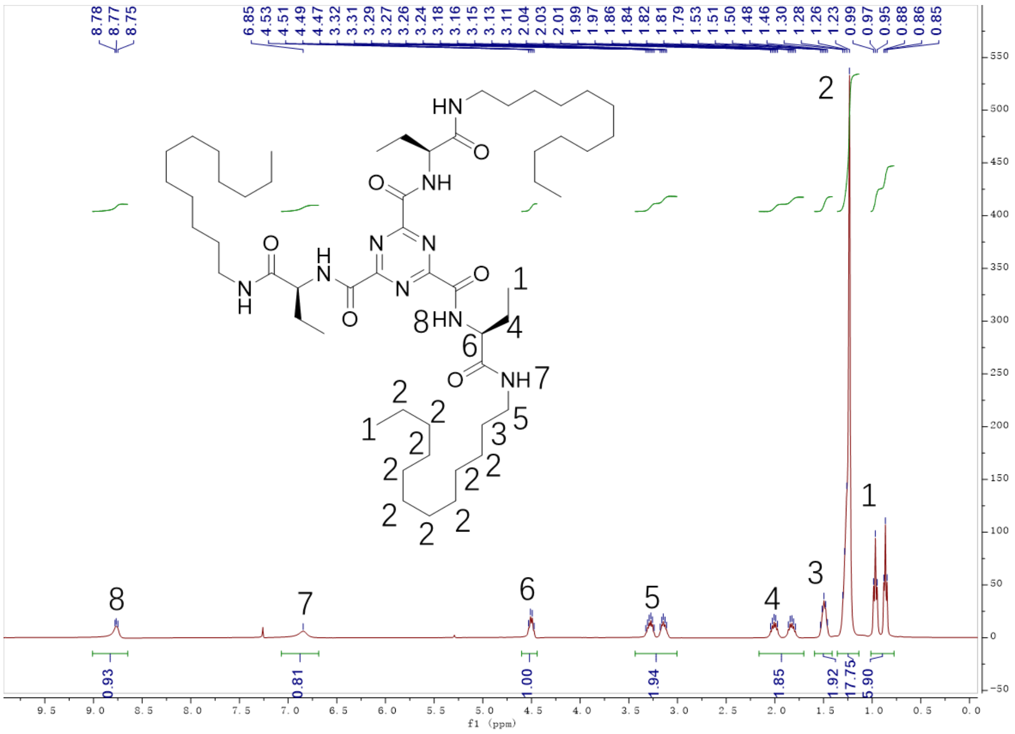

**Figure S33.** $^{1}$H NMR of **TTA 1** (400 MHz, 298 K, $CDCl_3$).

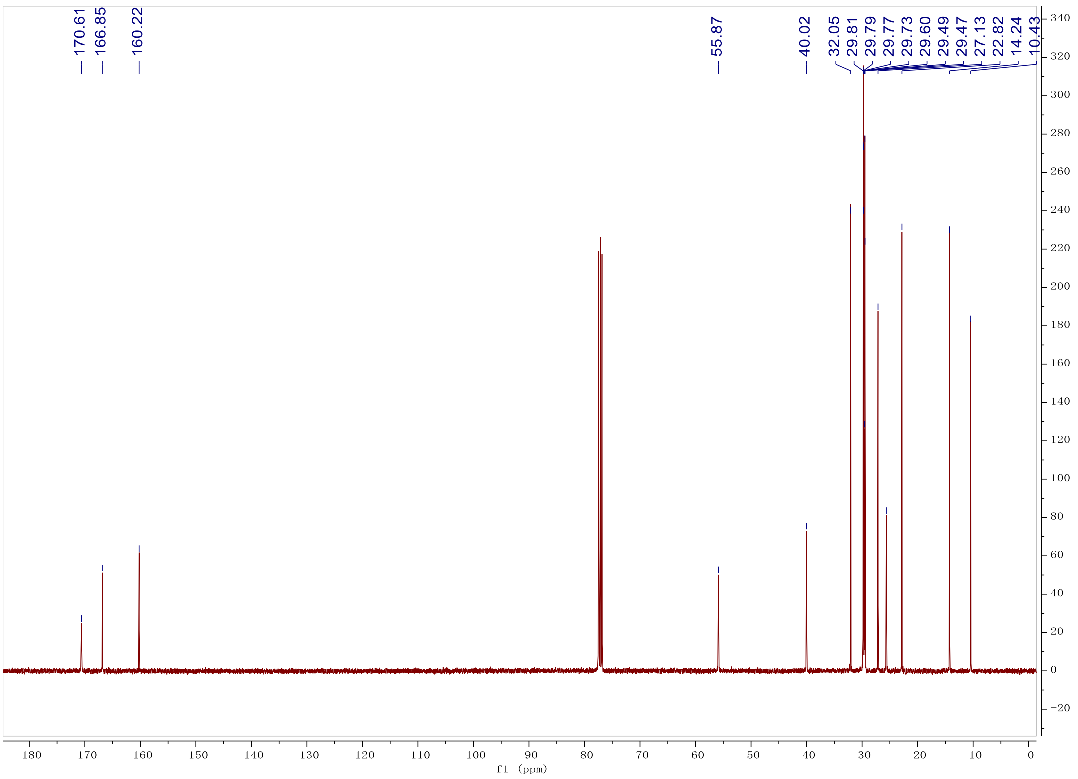


**Figure S34.** $^{13}$C NMR of **TTA 1** (101 MHz, 298 K, $CDCl_3$).

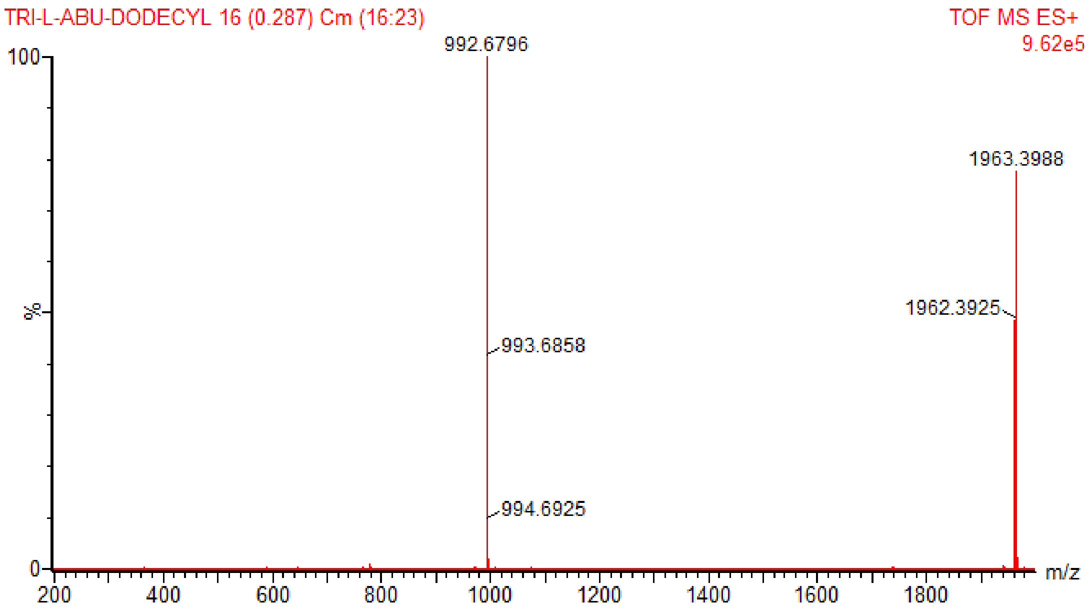


**Figure S35.** ESI-mass spectrum of **TTA 1** ($[M + Na]^+$ 992.6796).

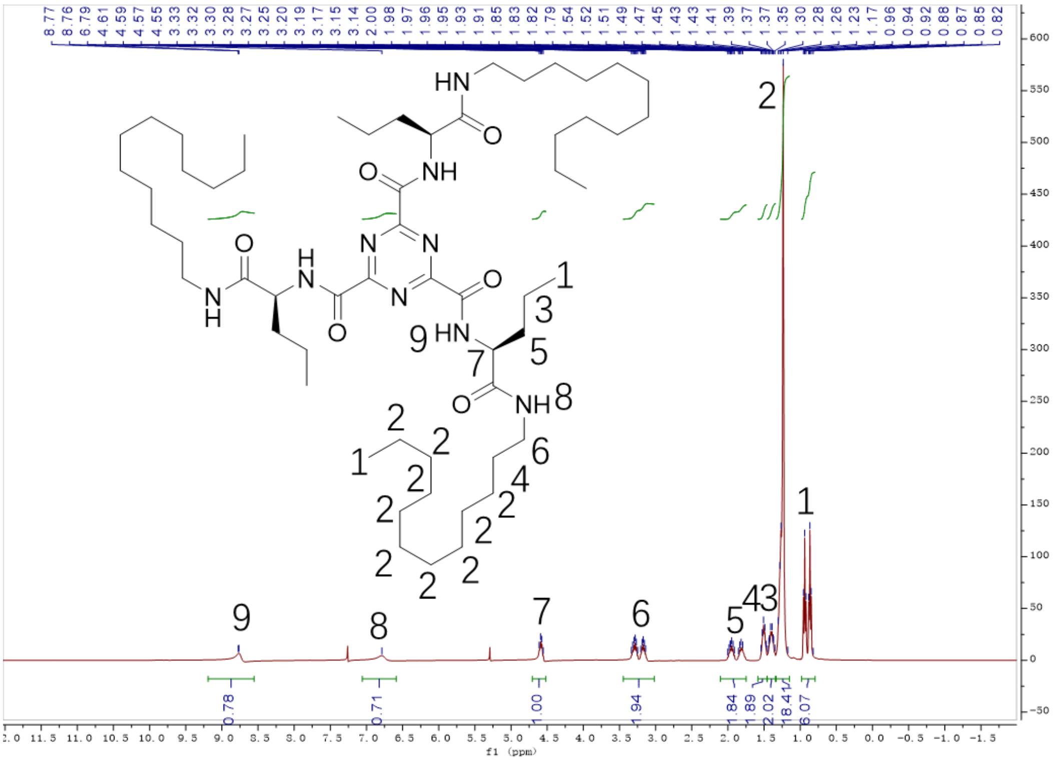


**Figure S36.** $^{1}$H NMR of **TTA 2** (400 MHz, 298 K, $CDCl_3$).

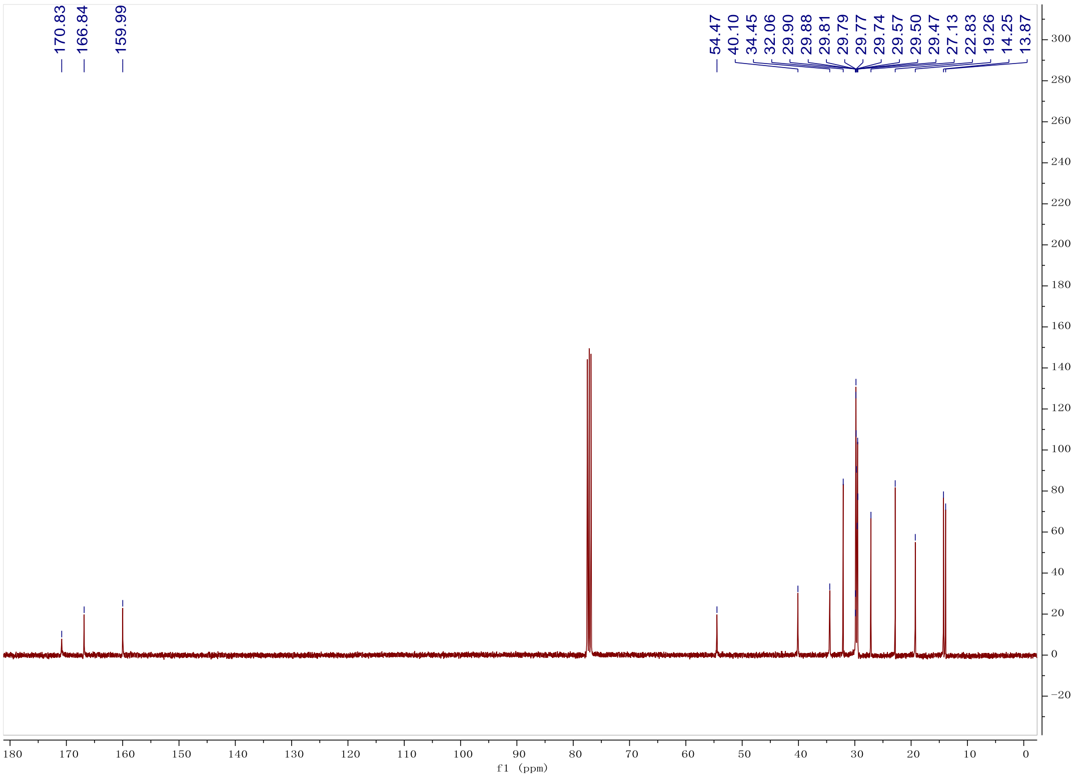


**Figure S37.** $^{13}$C NMR of **TTA 2** (101 MHz, 298 K, $CDCl_3$).

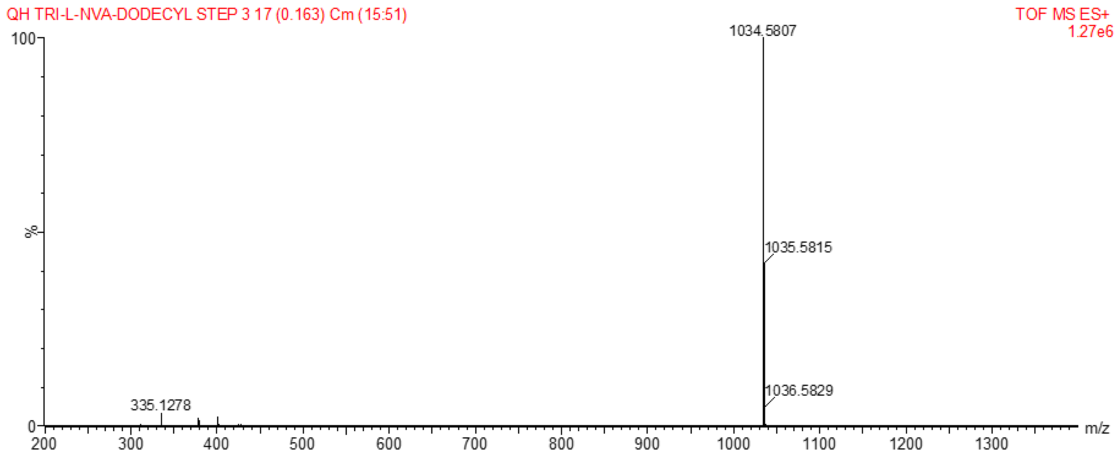


**Figure S38.** ESI-mass spectrum of **TTA 2** ([M + Na]+ 1034.5807).

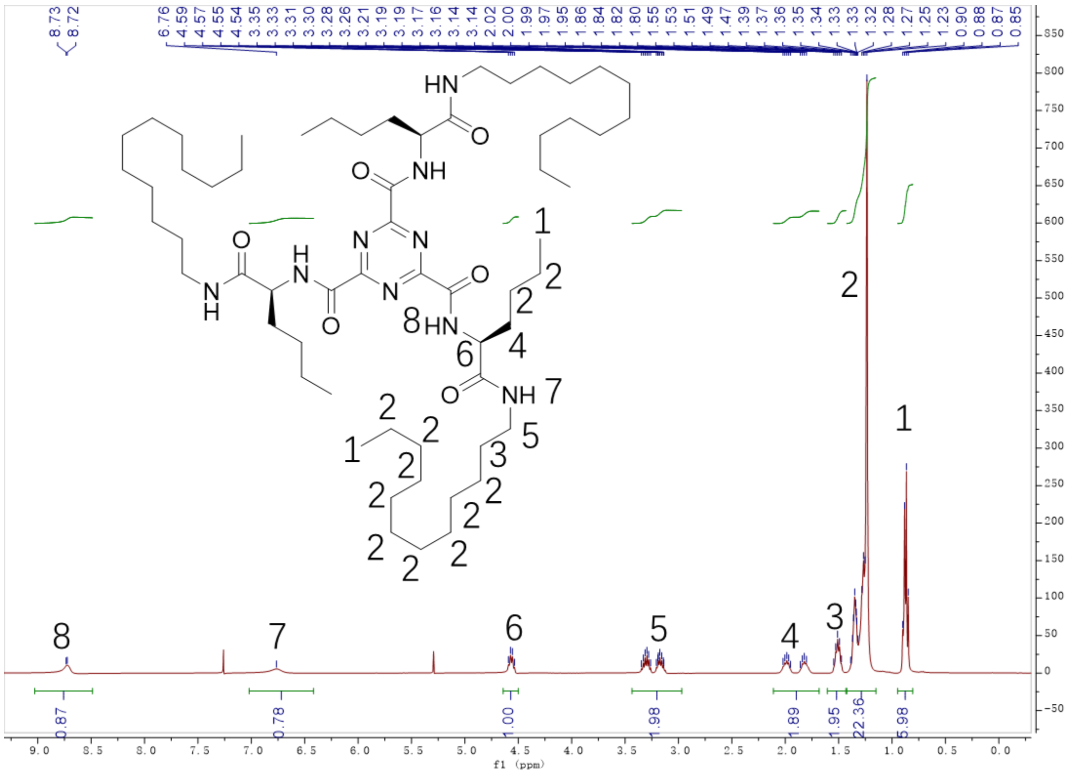

**Figure S39.** $^1$H NMR of **TTA 3** (400 MHz, 298 K, $CDCl_3$).

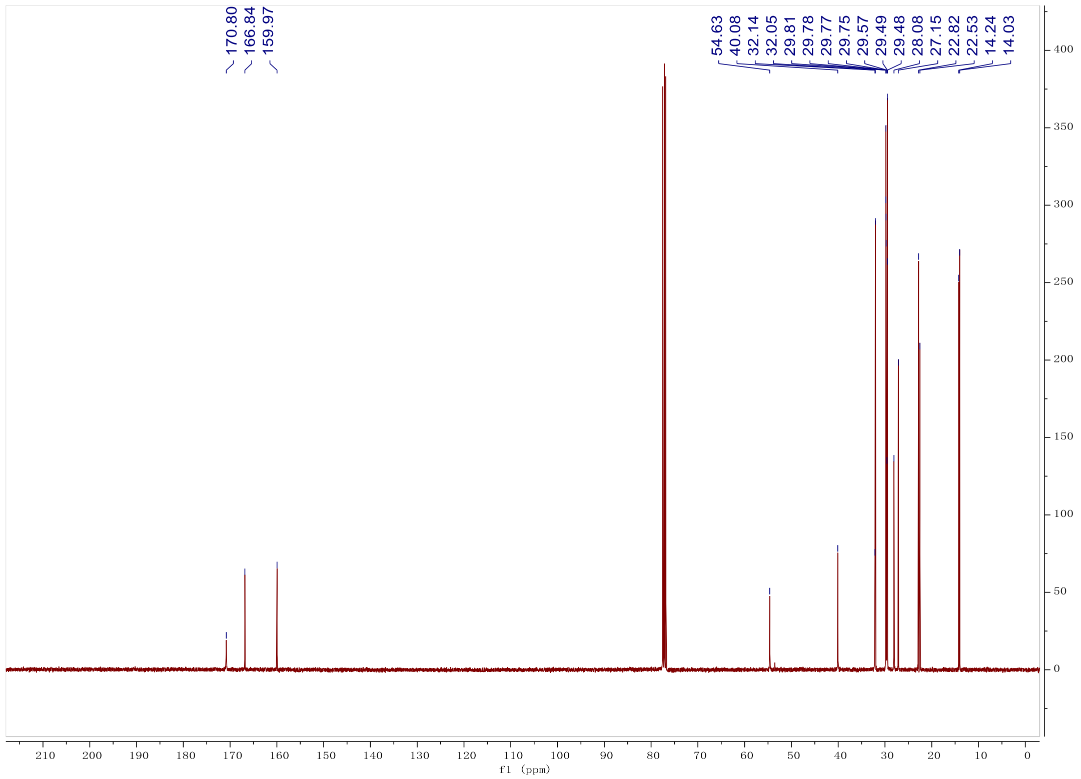


**Figure S40.** $^{13}C$ NMR of **TTA 3** (101 MHz, 298 K, $CDCl_3$).

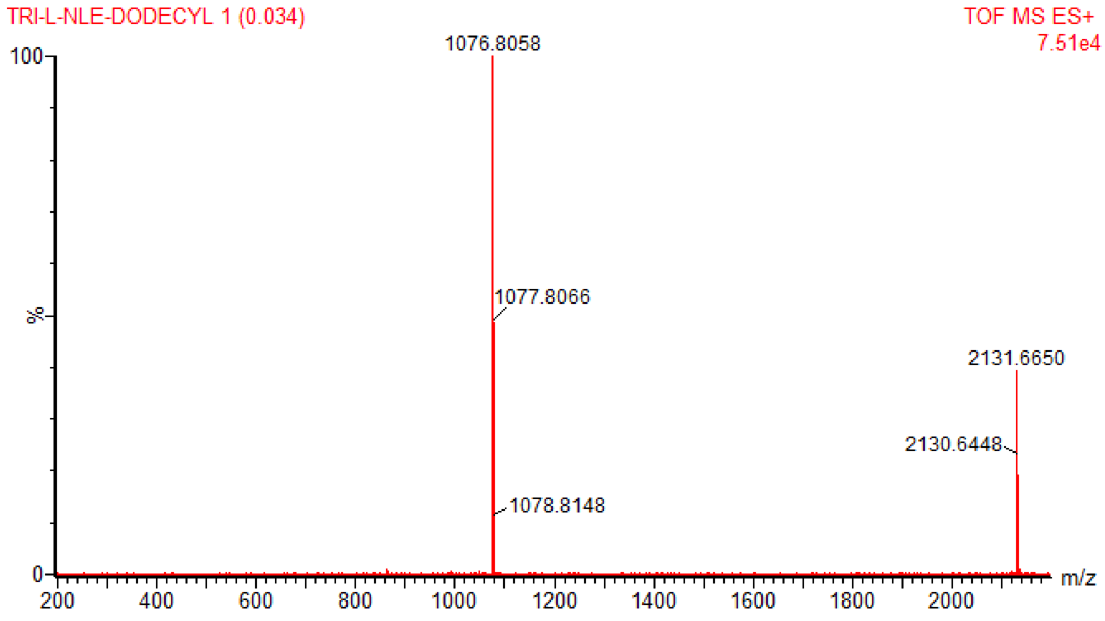


**Figure S41.** ESI-mass spectrum of **TTA 3** ($[M + Na]^+$ 1076.8058).

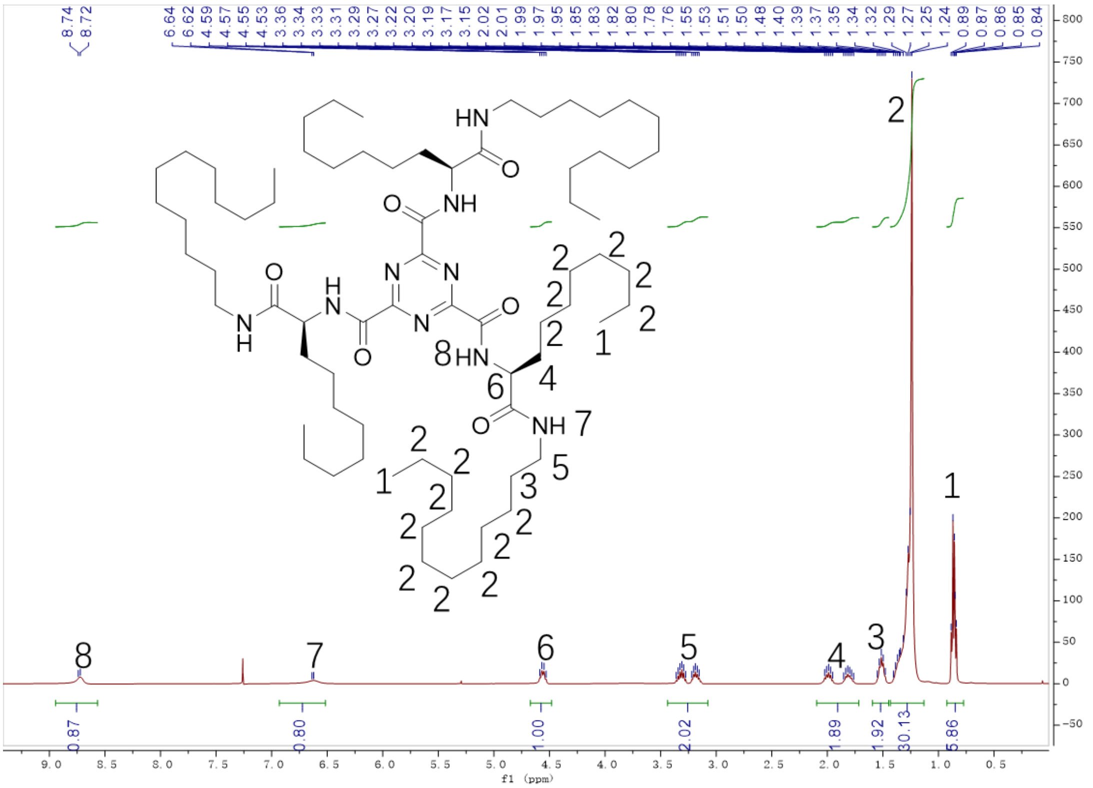

**Figure S42**. $^{1}$H NMR of **TTA 4** (400 MHz, 298 K, $CDCl_3$).

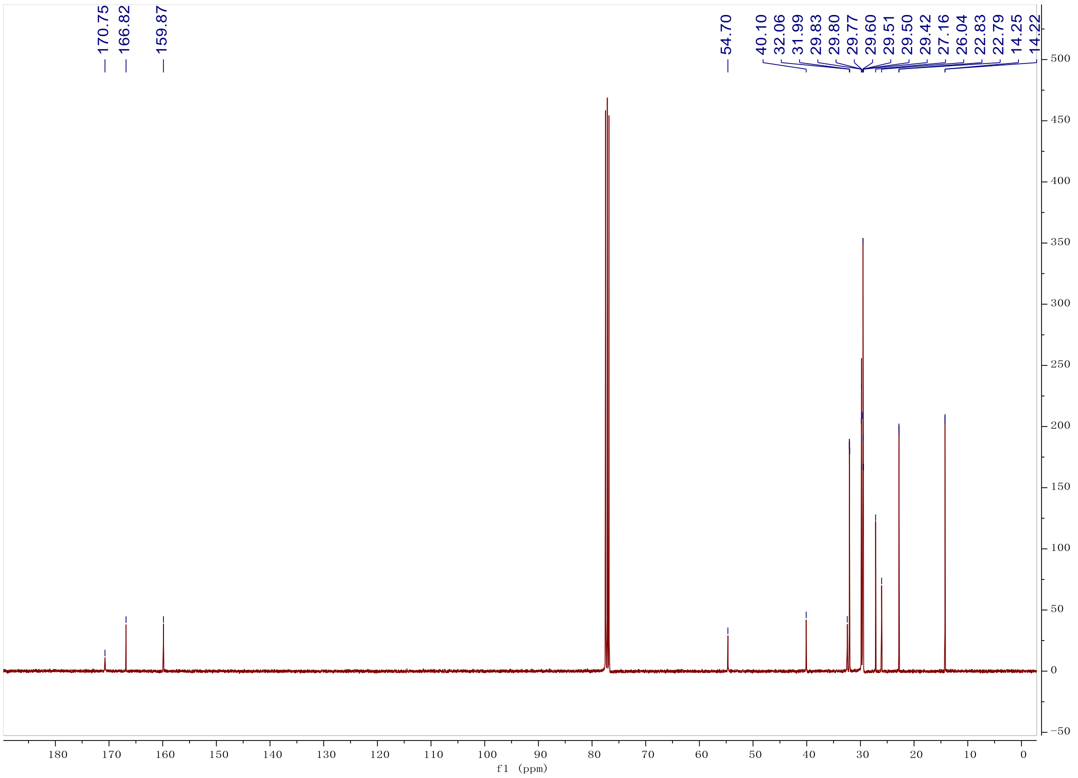

**Figure S43.** $^{13}$C NMR of TTA 4 (101 MHz, 298 K, $CDCl_3$).

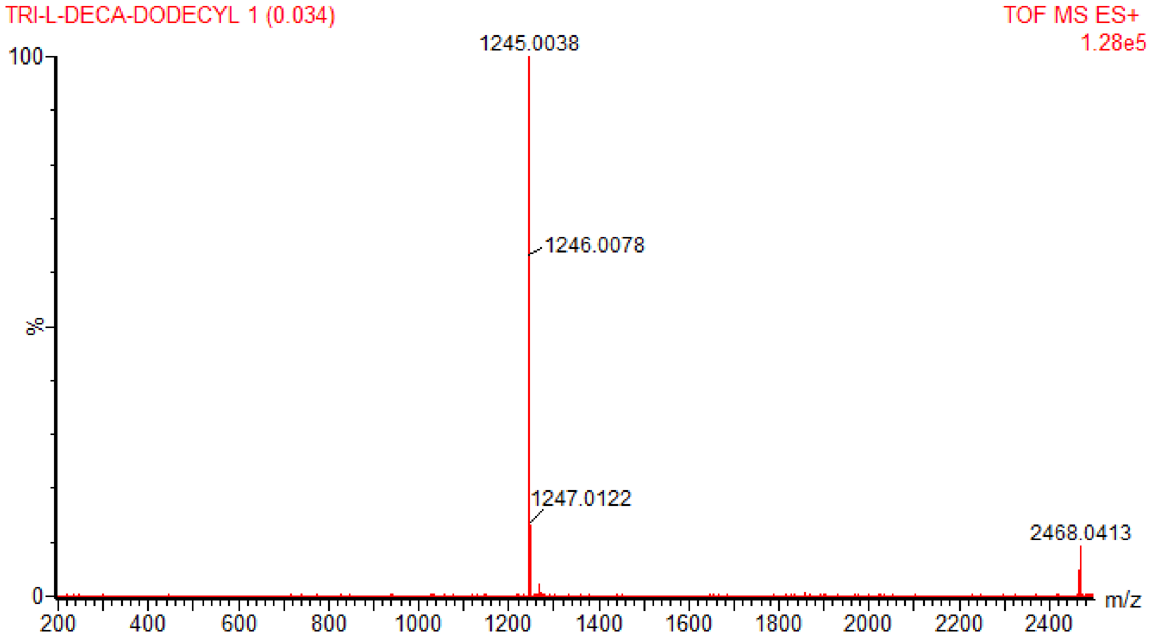


**Figure S44.** ESI-mass spectrum of **TTA 4** ($[M + Na]^+$ 1245.0038).

## 19. References


1. Matmin, J.; Yuliati, L.; Shamsuddin, M.; Lintang, H. O., Supramolecular Hydrogen Bonding Interactions of Novel 1,3,5-Benzenetricarbonyl Trisubstituted Alkyl for Anion Sensor Applications. *Adv. Mat. Res.* **2014,** *925*, 228-232.
2. Ogi, S.; Stepanenko, V.; Thein, J.; Würthner, F., Impact of Alkyl Spacer Length on Aggregation Pathways in Kinetically Controlled Supramolecular Polymerization. *J. Am. Chem. Soc.* **2016,** *138* (2), 670-678.
3. Smulders, M. M. J.; Schenning, A. P. H. J.; Meijer, E. W., Insight into the Mechanisms of Cooperative Self-Assembly: The "Sergeants-and-Soldiers" Principle of Chiral and Achiral C3-Symmetrical Discotic Triamides. *J. Am. Chem. Soc.* **2008,** *130* (2), 606-611.
4. Eden, K.; Morris, R.; Gillam, J.; MacPhee, Cait E.; Allen, Rosalind J., Competition between Primary Nucleation and Autocatalysis in Amyloid Fibril Self-Assembly. *Biophys. J.* **2015,** *108* (3), 632-643.
5. Grundmann, C.; Kober, E., Triazines. XVII. s-Triazine from s-Triazine-2,4,6-tricarbonic Acid. *J. Org. Chem.* **1956,** *21* (12), 1392-1394.
